# Intelligent Dental Handpiece: Real-Time Motion Analysis for Skill Development

**DOI:** 10.3390/s25206489

**Published:** 2025-10-21

**Authors:** Mohamed Sallam, Yousef Salah, Yousef Osman, Ali Hegazy, Esraa Khatab, Omar Shalash

**Affiliations:** 1 College of Artificial Intelligence, Arab Academy for Science, Technology and Maritime Transport, Alamin 51718, Egypt; m.h.sallam1@student.aast.edu (M.S.); y.s.semary@student.aast.edu (Y.S.); yousef.osman@student.aast.edu (Y.O.); alihegazy456@student.aast.edu (A.H.); 2 School of Mathematical and Computer Sciences, Heriot-Watt University, Dubai P.O. Box 501745, United Arab Emirates; e.khatab@hw.ac.uk; 3 Artificial Intelligence Research Center (AIRC), College of Engineering and Information Technology, Ajman University, Ajman P.O. Box 346, United Arab Emirates

**Keywords:** dental skill assessment, intelligent dental handpiece, real-time feedback, motion classification

## Abstract

Modern dental education increasingly calls for smarter tools that combine precision with meaningful feedback. In response, this study presents the Intelligent Dental Handpiece (IDH), a next-generation training tool designed to support dental students and professionals by providing real-time insights into their techniques. The IDH integrates motion sensors and a lightweight machine learning system to monitor and classify hand movements during practice sessions. The system classifies three motion states: Alert (10°–15° deviation), Lever Range (0°–10°), and Stop Range (>15°), based on IMU-derived features. A dataset collected from 61 practitioners was used to train and evaluate three machine learning models: Logistic Regression, Random Forest, Support Vector Machine (Linear RBF, Polynomial kernels), and a Neural Network. Performance across models ranged from 98.52% to 100% accuracy, with Random Forest and Logistic Regression achieving perfect classification and AUC scores of 1.00. Motion features such as Deviation, Take Time, and Device type were most influential in predicting skill levels. The IDH offers a practical and scalable solution for improving dexterity, safety, and confidence in dental training environments.

## 1. Introduction

Dental procedures demand a high level of dexterity, accuracy, and experience. Therefore, preclinical dental education plays a crucial role in helping students develop essential motor skills prior to engaging directly in patient care [1]. During this important training phase, students usually train using conventional dental simulators, which have played an important role in dental education since 1894 [2,3]. Although there are many limitations to conventional simulators, they enhanced the training process of students over a broad range of standardized technical procedures using dental instruments [4]. They remained an essential tool for simulating diverse dental procedures, preparing students for real-world dental practice [5].

An advancement in dental simulators is haptic simulation, which provides realistic video, audio, and tactile feedback to improve fine motor skills while providing unlimited training time, leading to enhanced students performance [3,6,7].

Dental handpieces have been used by dentists for decades; however, the increased complexity of procedures and the need for enhanced clinical outcomes have highlighted the need for more advanced dental handpieces through integrating real-time feedback and performance monitoring and evaluation [8]. This could be reached through the integration of Artificial Intelligence (AI) techniques into dental instruments, leading to precision-driven feedback [9,10,11].

Recent advances in Machine Learning (ML) and Deep Learning (DL) have significantly enhanced the ability to analyze and classify human motion across domains such as healthcare, robotics, sports, and human–computer interaction. Techniques like Support Vector Machines (SVMs), Random Forests, and deep neural networks have been widely applied to inertial measurement unit (IMU) data for gesture recognition and motion classification [12,13]. For instance, CNN-LSTM and CNN-GRU architectures have demonstrated high accuracy in recognizing dynamic gestures from IMU signals [13]. Reinforcement learning approaches, such as deep Q-networks, have also been explored for hand gesture recognition using EMG-IMU data [14]. In wearable systems, real-time hand tracking using deep learning models like MobileNet-SSD has shown promising results in human-computer interaction applications [15]. These methods, although developed for various applications, share core principles: feature extraction, temporal modeling, and classification. These principles are directly transferable to dental training. The proposed work builds on these foundations by applying and comparing multiple ML models for classifying dental handpiece motion states using IMU data.

Different commercial haptic dental handpieces are currently being used, as summarized in Table 1 [16]. Simondont is the most widely adopted solution offering immersive virtual reality and high-fidelity haptic feedback for a broad range of dental procedures. On the other hand, newer solutions, such as Virtual Reality Dental Training System (VRDTS) and Intelligent Dental Simulation System (IDSS), integrate advanced features such as AI-based assessment and real-time performance tracking. Notable IDSS emphasizes intelligent feedback and automated scoring [17,18].

Compared to the commercial systems mentioned in Table 1, the proposed Intelligent Dental Handpiece (IDH) offers several unique advantages. Unlike haptic-based simulators, the IDH focuses on real-time motion classification using IMU data and machine learning models, enabling lightweight and cost-effective feedback. IT supports cloud-based analytics, provides immediate visual and auditory alerts, and is built using compact, low-cost components. Furthermore, the IDH is accompanied by a publicly available dataset collected from 61 practitioners, supporting reproducibility and further research-related features that are rarely offered by commercial tools. These characteristics make the IDH a scalable and accessible solution for dental skill assessment and training.

In [27], authors presented a virtual system for training (hapTEL) using haptic technology. It allowed students to practice various procedures such as caries removal, dental drilling, and cavity preparation. Their prototype consisted of a PC, a Planar 3D stereo display, a camera head along with a Falcon haptic device, and a modified dental handpiece.

In [28], the authors introduced a robotic system guided by vision to improve spatial positioning accuracy during dental procedures. It integrated an enhanced force-feedback system and real-time visual guidance.

Multiple review papers have evaluated the acceptability and evaluation of haptic and virtual dental solutions. In [29], the authors studied evolution in the performance of learners trained to prepare access cavities incorporating Simodont, and also they determined learner acceptability of the Haptic Virtual Reality simulator (HVRS). Their results showed that it is reasonable to use Simodont, which was acceptable to learners for endodontic access cavity training. In [30], the authors examined the use of virtual assessment tools within the context of dental education in studies between 2000 and 2024. Their conclusion exploits how the virtual assessments have enhanced the accuracy and comprehensiveness of dental student evaluations; on the other hand, they highlighted some important challenges, such as the need for significant investment in technology, infrastructure, and training.

This research introduces the Intelligent Dental Handpiece (IDH), a novel integration of embedded sensor technology, real-time data processing, and smart feedback mechanisms into a conventional dental handpiece. Unlike traditional tools, the IDH captures motion and behavioral data during use, providing haptic and visual cues that alert the user to deviations in technique. These features aim not only to enhance procedural precision but also to support skill development and supervised learning, making it particularly valuable for dental education and training environments.

This paper presents the technical architecture, functionalities, and use-case scenarios of the IDH. It further evaluates its potential to transform current practices by enhancing the accuracy, accountability, and quality assurance in dental procedures. The work aims to lay a foundation for intelligent, data-driven systems within clinical dentistry, ultimately advancing both patient safety and practitioner development.

## 2. Prototype Design

**Figure 1:**
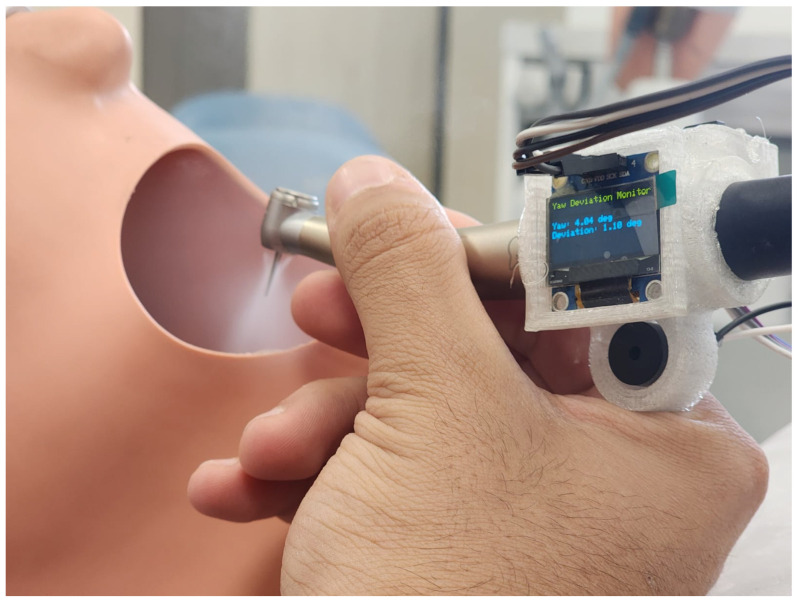
Intelligent dental handpiece in practice on phantom head model of a dental manikin.

**Figure 2:**
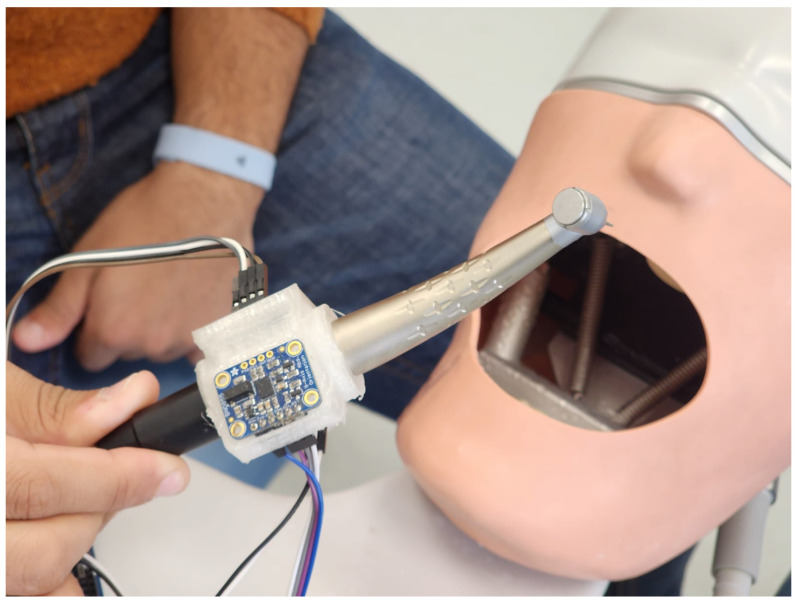
Another angle showing the intelligent dental handpiece in practice on phantom head model of a dental manikin.

The system consists of a dental handpiece with a 3D-printed compartment to mount some components installed to track and measure the movement of the students in practice. Figure 1 and Figure 2 showcase a dental training setup designed to track the movement of a dental handpiece in a simulated clinical setting. The device captures sensor data at a sampling rate of one sample per second. Each motion segment is evaluated as a short time series comprising consecutive sensor readings. To precisely identify the start and end of each movement, a manual trigger mechanism is employed: the practitioner activates a control button on the handpiece to mark the onset of the motion, and the release of the button designates the motion’s conclusion.

The device motion is tracked by IMU LSM6DS3TR-C -the micro-controller was manufactured by STMicroelectronics- Geneva, Switzerland) and transmitted to a cloud server to be processed by the developed machine learning model. The prototype components are as follows:

1. Air-turbine drill handpiece manufactured by NSK (Nakanishi Inc., Tochigi, Japan);
2. Microcontroller: XIAO nRF52840 manufactured by XIAO (Birmingham, UK);
3. IMU: LSM6DS3TR-C manufactured by XIAO STMicroelectronics (Plan-les-Ouates, Switzerland);
4. OLED: 0.96-inch SSD1306 (128×64 resolution) manufactured by Solomon Systech (Hong Kong, China);
5. Buzzer;
6. On/Off switch;
7. PCB.

**Figure 3:**
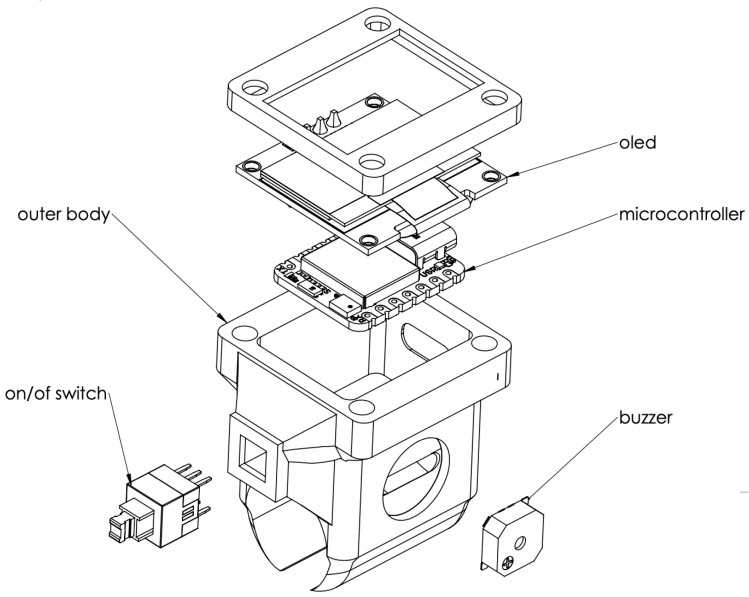
The developed AI-based device main compartment components.

The schematic design of the 3D compartment can be seen in Figure 3. The setup is being tested on a dental manikin, commonly used for training and practice, creating a realistic environment for evaluating hand control and technique. By capturing real-time motion data, this system could help assess skill levels, improve training methods, and even contribute to advancements in robotic-assisted dentistry. Ultimately, this kind of technology has the potential to refine how dental students and professionals develop precision and dexterity in their work.

**Figure 4:**
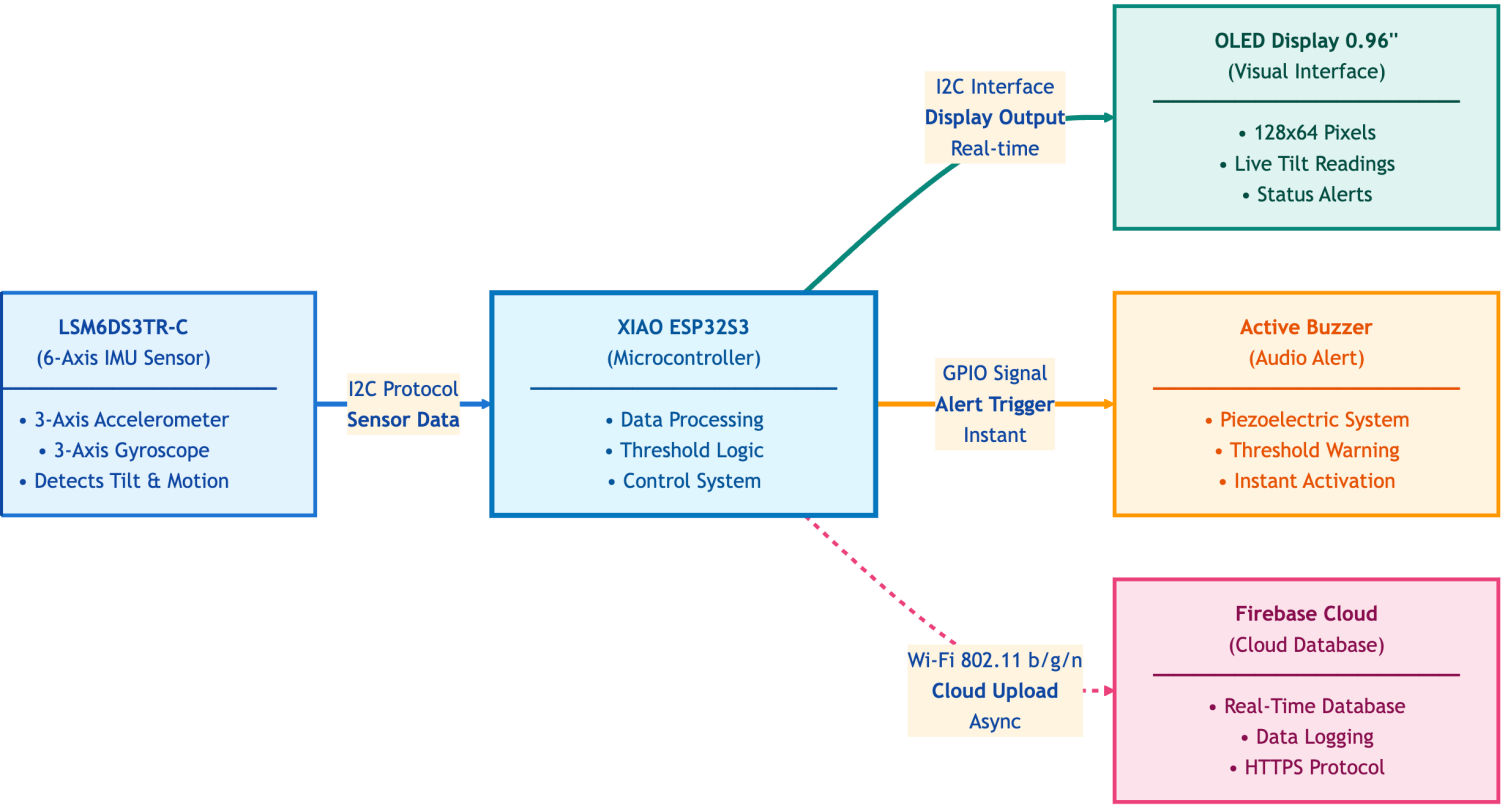
System diagram.

The system architecture in Figure 4 of the Intelligent Dental Handpiece is designed to seamlessly integrate motion sensing, real-time feedback, and cloud-based analytics into a compact and user-friendly setup. At its core, the LSM6DS3TR-C sensor captures six degrees of motion—acceleration and angular velocity—transmitting data via I^2^C to the XIAO ESP32S3 microcontroller (XIAO, Birmingham, UK). This microcontroller processes the data, applies threshold logic, and coordinates responses across the system. A 0.96" OLED display provides live visual feedback, while an active buzzer delivers immediate audio alerts when deviations occur. To support long-term tracking and remote analysis, the system uploads motion data asynchronously to a Firebase cloud database using Wi-Fi. Together, these components create a responsive and intelligent training tool that enhances precision, awareness, and skill development in dental education.

The developed prototype was used to collect 3720 data records from 61 practitioners (29 male and 31 female), with an average age of 38. Data was collected during the operation of opening cavities for fillings. The dataset comprises three balanced classes based on handpiece deviation angles: Class 0 (Alert) with 1239 samples (33.3%) representing deviation angles between 10° and 15°, Class 1 (Lever Range) with 1265 samples (34.0%) representing deviation angles between 0° and 10°, and Class 2 (Stop Range) with 1216 samples (32.7%) representing deviation angles exceeding 15°. Each data record includes the following parameters: ID, State, Time, Deviation, roll, pitch, yaw, acceleration, and velocity.

Each practitioner (user) has a unique ID to store their data separately. The State parameter defines the motion range observed, with values corresponding to one of the following: Lever Range, Alert, or Stop Range. The Time parameter represents the timestamp at which each record was collected. The roll, pitch, and yaw parameters describe the rotation axes of the handpiece during the procedure. The acceleration and velocity parameters indicate the linear dynamics of the tool’s movement during operation.

**Figure 5:**
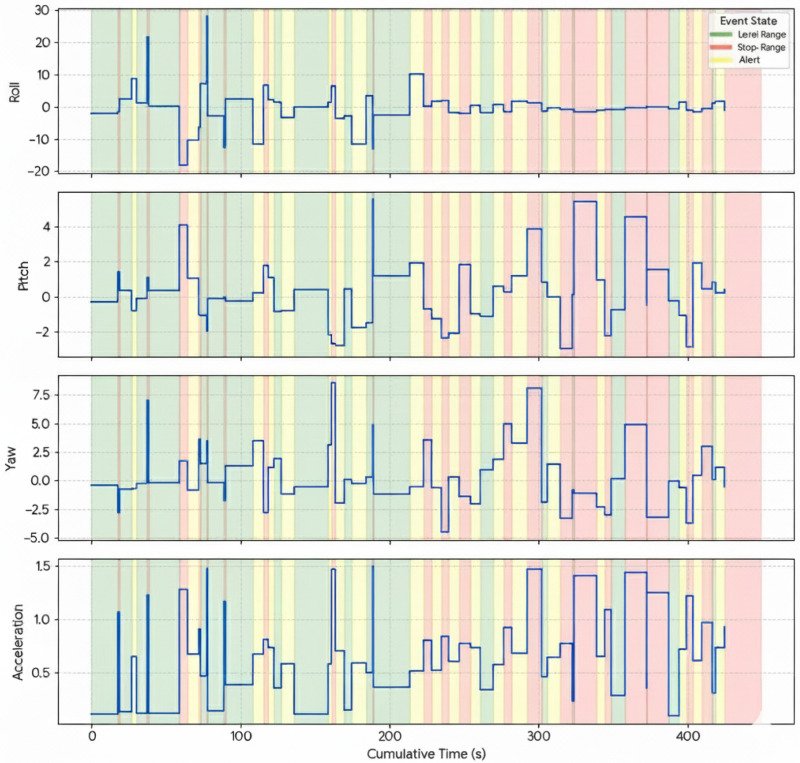
Visualization of collected signals for one practitioner showing roll, pitch, yaw, and acceleration over cumulative time. Background colors indicate motion states: green for Level Range, yellow for Alert, and red for Stop Range.

Figure 5 illustrates examples of the collected signals, where roll, pitch, yaw, and acceleration are plotted over time with color-coded background regions representing each state class (Level Range, Stop Range, and Alert). These visualizations help clarify the temporal and angular behavior of the handpiece across different states. More about the dataset is detailed in [31].

## 3. Methodology

This section presents the design and implementation of the proposed intelligent dental handpiece, focusing on data acquisition, preprocessing, and the motion analysis techniques.

The IMU is the main sensory component; it captures six degrees of freedom (DOF) of motion data, including acceleration, angular velocity, and orientation roll, pitch, and yaw, which are then analyzed to assess the dexterity and control. The following subsections elaborate on the preprocessing of IMU, feature extraction, ML models, and performance evaluation.

### 3.1. Data Preprocessing

The collection includes device-specific metadata together with data gathered by the IMU. Both numerical and categorical factors are present in these data. Deviation, Device, roll, pitch, yaw, acceleration, and velocity are the primary parameters in the datasets. The categorization category based on the manual observation made during data collection is represented by the target variable with the label State. Several preprocessing methods have been used to optimize the dataset for model training and evaluation.

#### 3.1.1. Feature Selection

The most crucial elements need to be found and extracted for model training and assessment. These features, which comprise device-specific data (Device and Deviation) and motion parameters (acceleration, roll, pitch, yaw, and velocity), were selected through exploratory analysis.

#### 3.1.2. Time Column Normalization

Every data record in the gathered dataset has a timestamp field. The time values are transformed to numerical values, counting the seconds since the recording started, to make numerical processing easier and guarantee consistent input ranges throughout the whole observations. The Min-Max scaling is then used to normalize them. The influence of temporal magnitude differences on model training was lessened by normalizing the results to a standard range of [0, 1] [32].

#### 3.1.3. Categorical Encoding

The dataset contains a State parameter in one of its columns. It is a categorical parameter that denotes various handpiece classes or operating states. Consequently, categorical values are transformed into numerical ones to guarantee compatibility with classification algorithms [33]. Without introducing ordinal bias, this modification preserved class information.

#### 3.1.4. Data Splitting

The train_test_split function from the scikit-learn library was used to divide the dataset into training and testing sections. A traditional 80/20 ratio was applied, with 20% of the data being used for performance evaluation and 80% being used to train the models. The random_state parameter was set to 42 in order to guarantee experiment repeatability.

### 3.2. Machine Learning Models

The analysis included a range of classifiers: Logistic Regression (LR), Random Forest (RF), the three main Support Vector Machine (SVM) kernels (Linear, RBF, Polynomial), and a Neural Network (NN) model. This mix covers both simple linear models and more complex deep-learning approaches, which helps identify the best pattern recognition method for the IMU sensor data. Each model was trained using the preprocessed data.

#### 3.2.1. Random Forest

An ensemble-based classifier, the Random Forest model builds several decision trees and combines their predictions [34]. It was selected for its inherent robustness against overfitting, its capacity to handle complex feature interactions, and its stability in analyzing high-dimensional, noisy sensor data.

#### 3.2.2. SVM (Kernel-Based)

The Support Vector Machine (SVM) classifier is highly regarded for its ability to locate the optimal separating hyperplanes between classes by maximizing the judgment margin. By explicitly exploring the Linear, Radial Basis Function (RBF), and Polynomial kernels, the study ensured that the best decision boundary—from a simple hyperplane to a complex, non-linear one—could be identified [35,36].

#### 3.2.3. Logistic Regression

A linear classifier that models the likelihood of an outcome, Logistic Regression was included as a highly interpretable performance benchmark [37,38]. It is crucial for assessing how much of the classification problem can be solved by simple linear relationships.

#### 3.2.4. Neural Network (NN)

A Neural Network (MLP) was selected to leverage its capacity for deep feature learning and modeling highly non-linear, hierarchical patterns within the time-series sensor data. NN models provide a state-of-the-art benchmark, particularly when complex spatial or temporal dependencies exist in motion data [39,40].

### 3.3. Hyperparameter Optimization Framework

Hyperparameter tuning was performed using Randomized Search with stratified k-fold cross-validation to optimize model performance and generalization. Randomized search efficiently explores vast hyperparameter spaces by randomly sampling from specified distributions, in contrast to exhaustive grid search, which analyzes every possible combination [41]. This method is particularly advantageous when computational resources are limited or when certain parameters have minimal performance impact [42].

#### 3.3.1. Randomized Search Methodology

Randomized search was selected over exhaustive grid search based on empirical studies demonstrating comparable performance with significantly reduced computational cost [41]. By randomly sampling from specified parameter distributions rather than exhaustively evaluating every combination, this method efficiently explores vast hyperparameter spaces while maintaining optimization quality. This approach is particularly advantageous when computational resources are limited or when certain parameters have minimal performance impact [42].

The randomized search process samples hyperparameter combinations from predefined distributions, evaluating each configuration through cross-validation to estimate generalization performance. The number of search iterations was determined based on each model’s parameter space complexity, balancing thorough exploration with computational feasibility. Model-specific iteration counts ranged from 15 to 30, as detailed in Table 2.

#### 3.3.2. Cross-Validation Strategy

All models employed 5-fold stratified cross-validation to ensure reliable performance estimation and prevent overfitting [43]. Stratification maintains the original class distribution within each fold, which is critical for imbalanced datasets and ensures that model performance estimates are representative across all classes.

General Configuration:

- Cross-Validation Method: 5-fold stratified cross-validation (k=5);
- Primary Optimization Metric: classification accuracy;
- Secondary Metrics: precision, recall, and F1-score (monitored for balanced performance);
- Random State: fixed at 42 for full reproducibility;
- Training/Test Split: 80%/20% with stratified sampling.

The optimization workflow consists of the following:

1. Split dataset into training (80%) and test (20%) sets with stratification.
2. For each hyperparameter combination sampled
  (a) Divide training set into five stratified folds;
  (b) Train model on four folds, validate on the remaining fold;
  (c) Rotate validation fold and repeat (five iterations total);
  (d) Calculate mean and standard deviation of accuracy across folds.
3. Select hyperparameters yielding highest mean cross-validation accuracy.
4. Retrain model on entire training set with optimal parameters.
5. Evaluate final model on held-out test set.

Table 2 summarizes the search configuration for each model, including the number of iterations, cross-validation folds, parameters tuned, and total model evaluations performed.

#### 3.3.3. Model-Specific Parameter Search Spaces

For each model, hyperparameter distributions were carefully specified based on established best practices and theoretical considerations [44]. The following subsections detail the search space for each algorithm.

##### Logistic Regression

Search iterations: 20.

Parameter distributions:

- C (Inverse Regularization Strength): Log-uniform distribution $U_{\log}\left[0.01,100\right]$;
- Penalty Type: Categorical {L1, L2};
- Solver: Categorical {liblinear, lbfgs, saga}.

Optimal configuration: C=78.53, penalty = L2, solver = lbfgs.

##### Random Forest

Search iterations: 30.

Parameter distributions:

- n_estimators: Discrete uniform $U_{\mathrm{int}}\left[100,500\right]$;
- max_depth: Discrete uniform $U_{\mathrm{int}}\left[5,30\right]$;
- min_samples_split: Discrete uniform $U_{\mathrm{int}}\left[2,20\right]$;
- min_samples_leaf: Discrete uniform $U_{\mathrm{int}}\left[1,10\right]$;
- max_features: Continuous uniform U[0.3,1.0].
- criterion: Categorical {gini, entropy}.

Optimal configuration: n_estimators = 221, max_depth = 24, min_samples_split = 8, min_samples_leaf = 8, max_features = 0.966, criterion = gini.

##### Support Vector Machine—Linear Kernel

Search iterations: 15

Parameter distributions:

- C: Log-uniform $U_{\log}\left[0.01,100\right]$.
- class_weight: Categorical {None, balanced}

Optimal configuration: C=21.37, class_weight = balanced.

##### Support Vector Machine—RBF Kernel

Search iterations: 20.

Parameter distributions:

- C: Log-uniform $U_{\log}\left[0.1,100\right]$;
- gamma: Log-uniform $U_{\log}\left[0.001,1\right]$;
- class_weight: Categorical {None, balanced}.

Optimal configuration: C=65.41, gamma = 0.0035, class_weight = balanced.

##### Support Vector Machine—Polynomial Kernel

Search iterations: 20.

Parameter distributions:

- C: Log-uniform $U_{\log}\left[0.1,100\right]$;
- degree: Discrete uniform $U_{\mathrm{int}}\left[2,5\right]$;
- gamma: Log-uniform $U_{\log}\left[0.001,1\right]$;
- coef0: Continuous uniform U[0,1];
- class_weight: Categorical {None, balanced}.

Optimal configuration: C=34.17, degree = 2, gamma = 0.0576, coef0 = 0.271, class_weight = None.

##### Neural Network

Unlike the conventional machine learning models, the Neural Network architecture was chosen based on established literature best practices for this data type and was held fixed to ensure a fair comparison against the tuned classical models without requiring an expensive, separate hyperparameter search.

Fixed Configuration:

- Architecture: two hidden layers with [64, 32] neurons;
- Activation Function: ReLU [45];
- Dropout Rate: 20% [46];
- Optimizer: Adam with learning rate α=0.001 [47];
- Loss Function: sparse categorical cross-entropy (multiclass);
- Early Stopping: patience = 10 epochs [48];
- Batch Size: 32 samples;
- Maximum Epochs: 100.

## 4. Experiments

This section describes the computational environment and evaluation framework used to evaluate the performance of different classification models.

### 4.1. Experimental Setup

An OMEN by HP Laptop 15-dh1xxx personal computer (HP Inc., Palo Alto, CA, USA) with an Intel(R) Core(TM) i7-10750H CPU @ 2.60GHz, 16.0 GB of RAM, and an NVIDIA GeForce GTX 1660 Ti (6 GB) graphics card (NVIDIA Corporation, Santa Clara, CA, USA) was used for all trials. Windows 64-bit (Microsoft Corporation, Redmond, WA, USA) was the operating system utilized. Python 3.9.7 (Python Software Foundation, Wilmington, DE, USA) was used for model implementations, with scikit-learn 1.2.0, TensorFlow 2.11.0 (Google LLC, Mountain View, CA, USA), and XGBoost 2.1.4 (DMLC, distributed machine learning community) as essential libraries.

### 4.2. Performance Metrics

ROC AUC utilizing a One-vs.-Rest (OvR) technique, test accuracy, weighted precision, weighted recall, weighted F1-score, and other common evaluation metrics were used to evaluate the predictive validity of the classification models [49].

By computing the percentage of properly predicted cases out of all the instances in the test set, test accuracy [50] measures the model’s overall correctness. Simple and easy to use, it could not provide as much information in datasets that are unbalanced. Calculated each class and then averaged depending on class frequency, weighted precision [51] assesses the percentage of accurate positive predictions among all positive predictions generated by the model. This measure is especially crucial in situations when false positives are expensive.

The percentage of genuine positive predictions among all actual instances of each class is measured by weighted recall [51], which is also combined using class-weighted averaging. In situations where false negatives are more important, this statistic is particularly pertinent.

The weighted F1-score [52] uses the harmonic mean of precision and recall to integrate them into a single metric. By weighting by the amount of true cases in each class, it corrects for class imbalance and takes into consideration both false positives and false negatives.

Last but not least, the model’s capacity to differentiate between classes over a range of classification thresholds is evaluated using the ROC AUC (Area Under the Receiver Operating Characteristic Curve) utilizing a One-vs.-Rest (OvR) strategy [50]. The model’s discriminative strength is revealed in the multiclass environment by reporting a weighted average of the AUC scores for every class.

## 5. Results and Discussion

This section provides a detailed performance evaluation and interpretation of multiple classification models, including Linear Support Vector Machine (SVM), Logistic Regression, Random Forest, and other kernel-based methods. The assessment utilizes a range of quantitative metrics and visualization techniques.

### 5.1. Linear SVM Classifier Performance Analysis

The motion data was successfully classified by the Linear Support Vector Machine (SVM) Classifier. Numerous assessment indicators and visualization tools demonstrate its efficacy.

**Figure 6:**
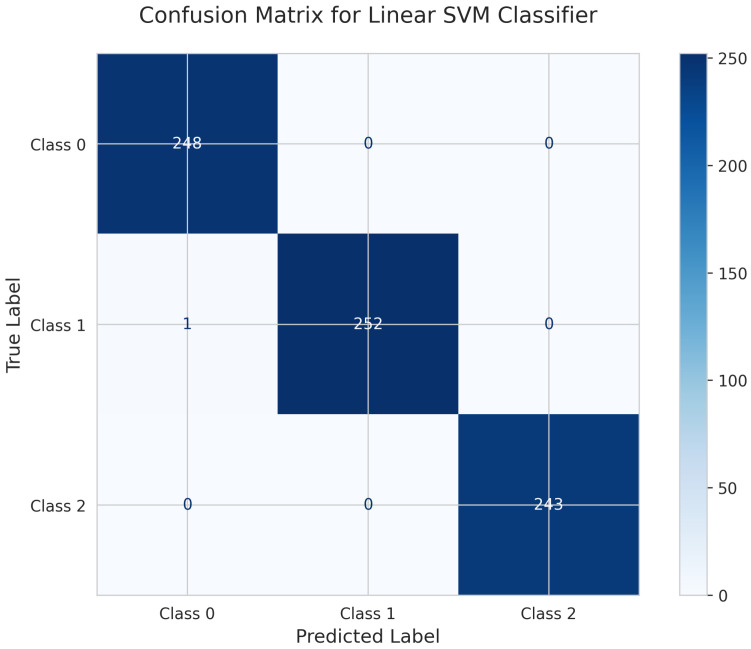
Confusion matrix for Linear SVM Classifier.

The confusion matrix for the Linear Support Vector Machine (SVM) Classifier (Figure 6) indicates high classification accuracy, with only one misclassification overall. For Class 0, all 248 instances were correctly classified. Class 1 achieved 252 true positives, with a single instance misclassified as Class 0. Class 2 had 243 true positives and no misclassifications. These results indicate that the Linear SVM model effectively distinguishes among the three motion classes. The test accuracy for the Linear SVM Classifier was 0.996, or 99.6%.

**Figure 7:**
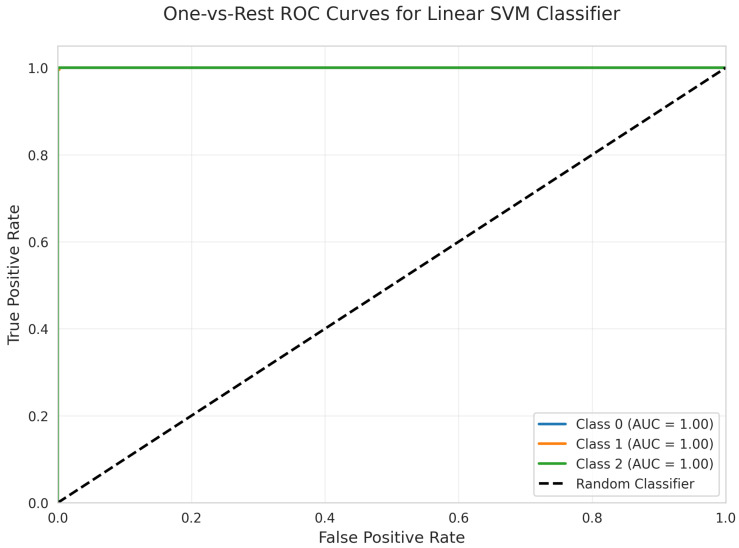
One -vs.-Rest ROC Curves for Linear SVM Classifier. All classes overlap at 100%.

The One-vs.-Rest Receiver Operating Characteristic (ROC) Curves (Figure 7) further confirm the model’s discriminative capacity. All three classes (0, 1, and 2) achieved perfect separation, each with an Area Under the Curve (AUC) of 1.00. These results indicate that the model consistently achieves optimal true positive rates and zero false positive rates across all classes.

**Figure 8:**
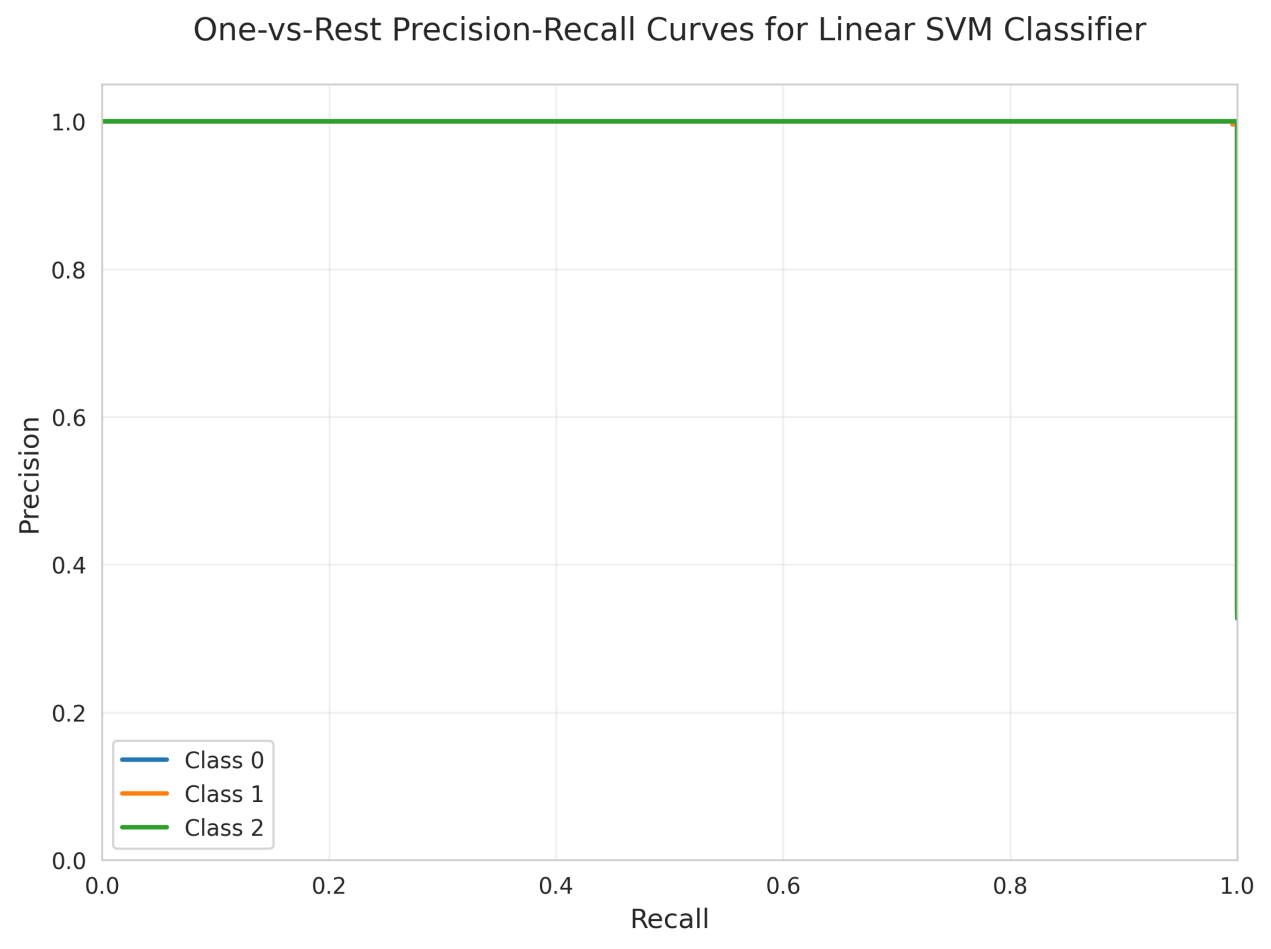
One-vs.-Rest Precision-Recall Curves for Linear SVM Classifier. All classes overlap at 100%.

The One-vs.-Rest Precision-Recall Curves (Figure 8) illustrate the model’s effectiveness in positive class prediction. All three classes maintain a precision of 1.00 across all recall values, indicating highly reliable positive class predictions. The weighted test F1-score, recall, and precision were each 0.996.

**Figure 9:**
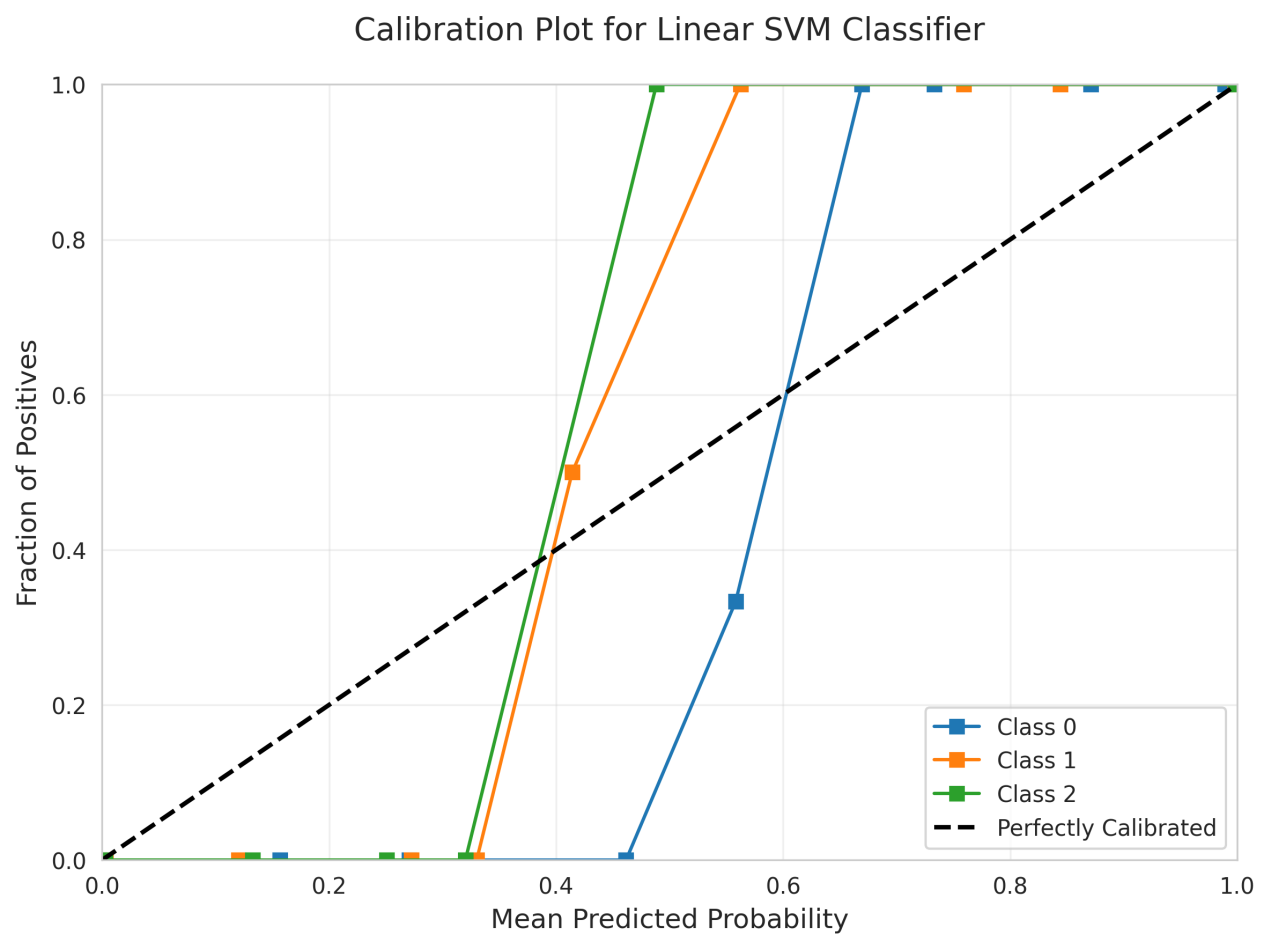
Calibration Plot for Linear SVM Classifier.

The Calibration Plot (Figure 9) evaluates the alignment between predicted and actual probabilities for the Linear Support Vector Machine (SVM) classifier. The curves for all three classes closely follow the diagonal line across all probability ranges, indicating that the predicted probabilities are highly reliable and well-calibrated. This level of calibration supports the model’s suitability for applications that require accurate probability estimates.

**Figure 10:**
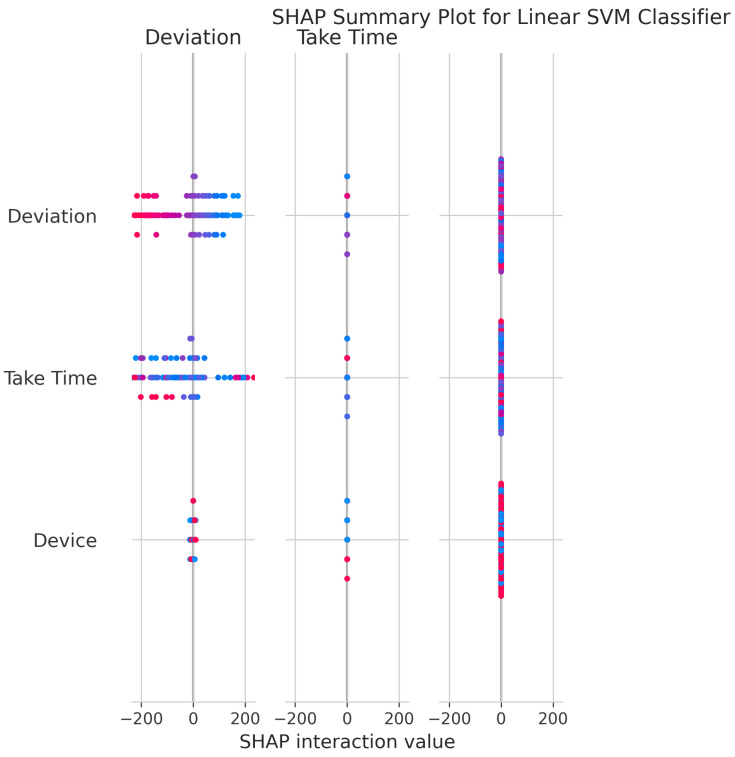
SHAP Summary Plot for Linear SVM Classifier. High valued features are represented in red, while low valued features are represented in blue.

The SHAP Summary Plot (Figure 10) provides insight into feature contributions to the model’s output. Deviation and Take Time have the greatest impact, as indicated by their broad ranges of SHAP values. The vertical spread and color-coding by feature value demonstrate how each feature’s magnitude influences predictions. Interaction values further confirm that Deviation and Take Time primarily exert direct effects on model decisions, rather than relying on complex interactions with other features.

The Linear SVM classifier, optimized using RandomizedSearchCV, achieved a cross-validation (CV) score of 0.999 ± 0.001 with the optimal hyperparameters: C=21.37 and class_weight = `balanced’. However, nine out of fifteen fitting attempts failed due to an unsupported combination of penalty = `l2’ and loss = `hinge’ when dual = False.

### 5.2. Logistic Regression Performance Analysis

The Logistic Regression model demonstrated exceptional performance, accurately capturing linear relationships present in the dataset.

**Figure 11:**
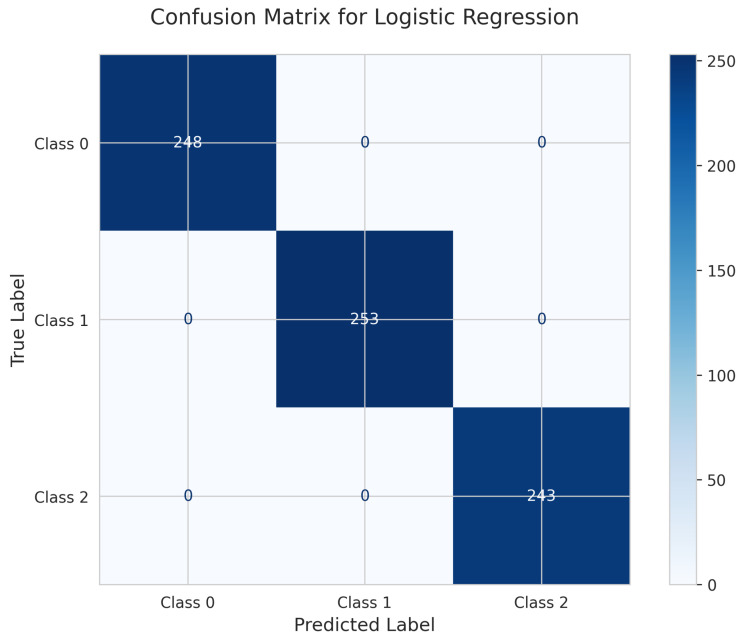
Confusion Matrix for Logistic Regression.

Perfect predictions are shown by the Logistic Regression Confusion Matrix (Figure 11). All 248 instances for Class 0, 253 instances for Class 1, and 243 instances for Class 2 were correctly identified with zero misclassifications. This demonstrates the exceptional effectiveness of the linear model on this dataset, achieving a perfect 100% test accuracy.

**Figure 12:**
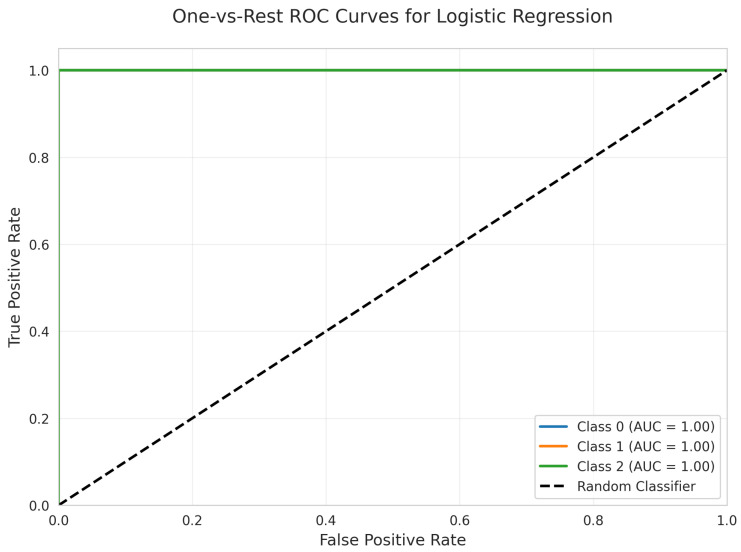
One-vs.-Rest ROC Curves for Logistic Regression. All classes overlap at 100%.

The One-vs.-Rest ROC Curves (Figure 12) for Logistic Regression demonstrate perfect discriminative performance, with a weighted ROC AUC of 1.00 for all classes. Each class achieves an AUC of 1.00, with curves located at the top-left corner, indicating optimal true positive rates and zero false positive rates.

**Figure 13:**
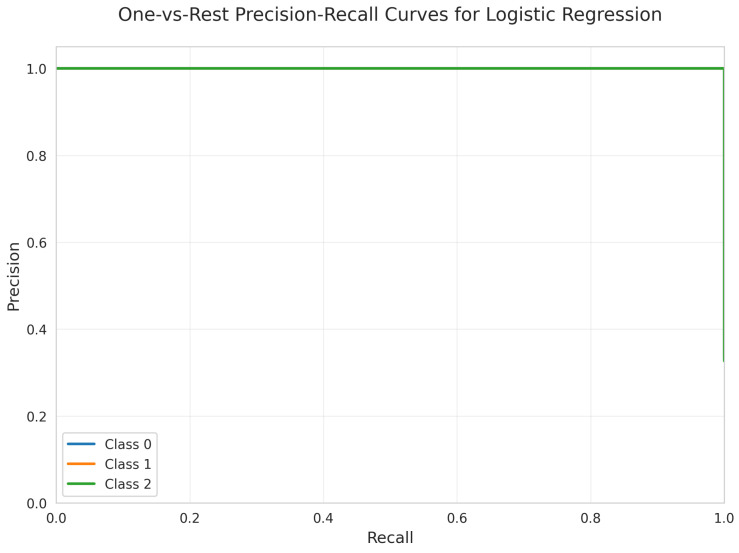
One-vs.-Rest Precision-Recall Curves for Logistic Regression. All classes overlap at 100%.

The One-vs.-Rest Precision-Recall Curves (Figure 13) for all three classes demonstrate outstanding precision and recall. Each class maintains a precision of 1.00 across all recall values, indicating highly effective classification. The weighted test precision, recall, and F1-score were each 1.000.

**Figure 14:**
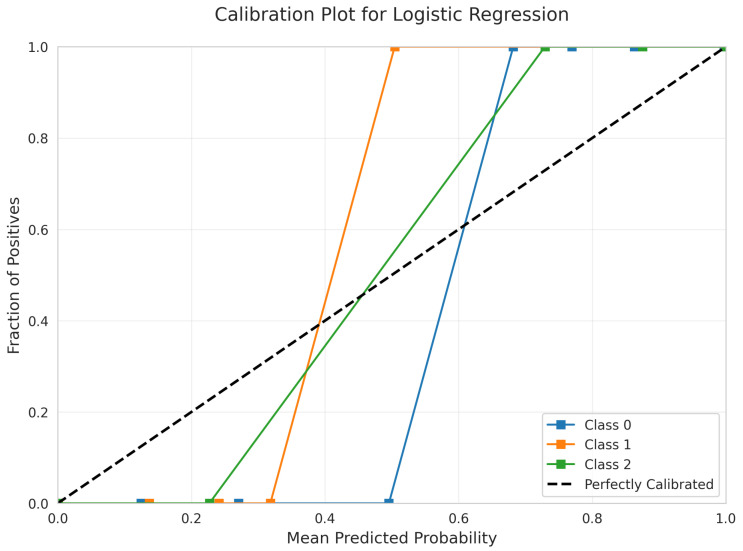
Calibration Plot for Logistic Regression.

The model’s predicted probabilities are exceptionally well-calibrated, according to the Calibration Plot (Figure 14) for Logistic Regression. The curves for all three classes closely follow the diagonal line across all probability ranges, meaning that predicted probabilities accurately reflect true class membership probabilities. This quality is highly desirable for applications where accurate probability estimates are essential.

**Figure 15:**
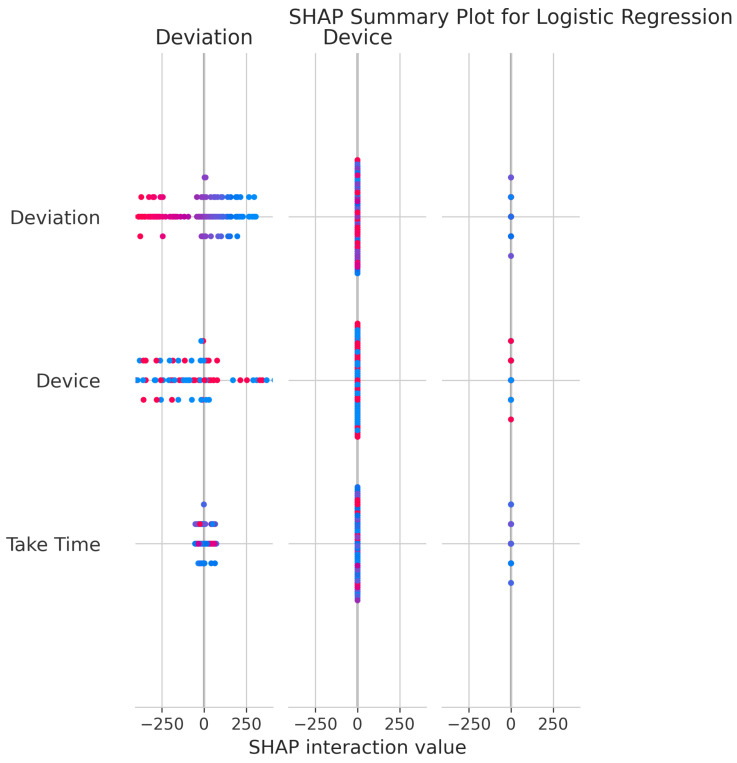
SHAP Summary Plot for Logistic Regression. High valued features are represented in red, while low valued features are represented in blue.

The SHAP Summary Plot for Logistic Regression (Figure 15) clarifies feature influence on model output. Deviation and Device exhibit the greatest effect, as indicated by their wide range of SHAP values. These features consistently exert strong influence on individual predictions, with higher values driving predictions in specific directions.

Logistic Regression, optimized using RandomizedSearchCV, achieved a cross-validation (CV) score of 0.9987 ± 0.0007 with the optimal hyperparameters: C=78.53, penalty = `l2’, and solver = `lbfgs’. During training and evaluation, a FutureWarning indicated that the multi_class = `multinomial’ setting will be adopted by default in future library versions.

### 5.3. Random Forest Classifier Performance Analysis

The Random Forest Classifier demonstrated exceptionally high performance, achieving perfect classification on this dataset.

**Figure 16:**
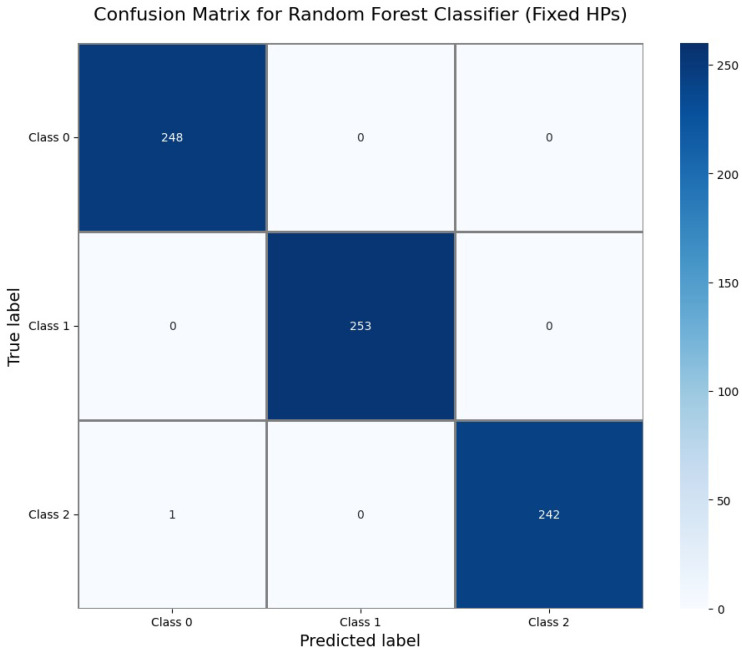
Confusion Matrix for Random Forest Classifier (fixed hyperparameters).

The Confusion Matrix for the Random Forest Classifier (Figure 16) demonstrates perfect classification. All 248 instances of Class 0, 253 of Class 1, and 243 of Class 2 were correctly identified, resulting in zero misclassifications and an overall test accuracy of 1.000.

**Figure 17:**
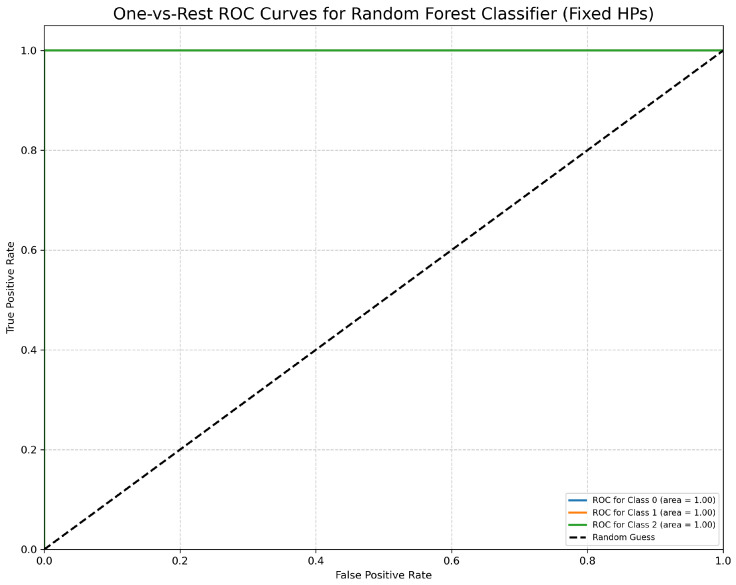
One-vs.-Rest ROC Curves for Random Forest Classifier (fixed hyperparameters). All classes overlap at 100%.

The One-vs.-Rest ROC Curves (Figure 17) further confirm the Random Forest Classifier’s discriminative capacity. All classes achieve an Area Under the Curve (AUC) of 1.00, indicating perfect separation between classes.

**Figure 18:**
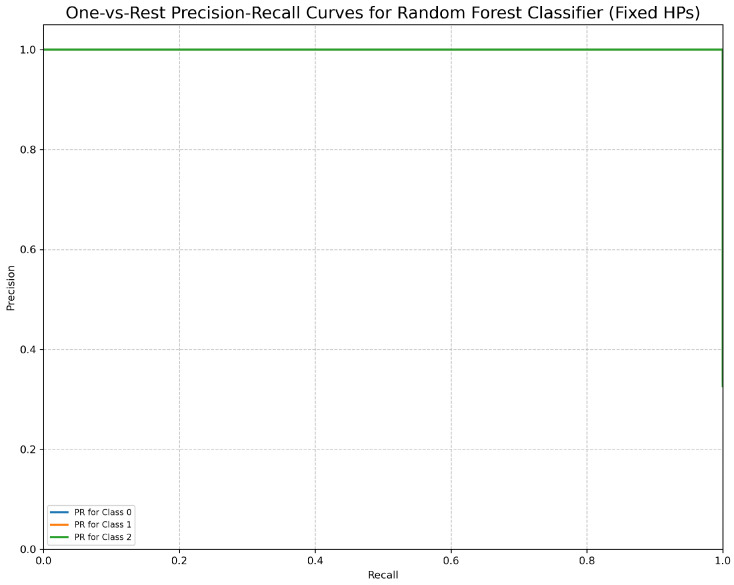
One-vs.-Rest Precision-Recall Curves for Random Forest Classifier (fixed hyperparameters). All classes overlap at 100%.

The One-vs.-Rest Precision-Recall Curves (Figure 18) for the Random Forest Classifier demonstrate flawless precision of 1.00 across all recall values for all classes. This result indicates perfect performance, with 100% precision maintained at all recall levels.

**Figure 19:**
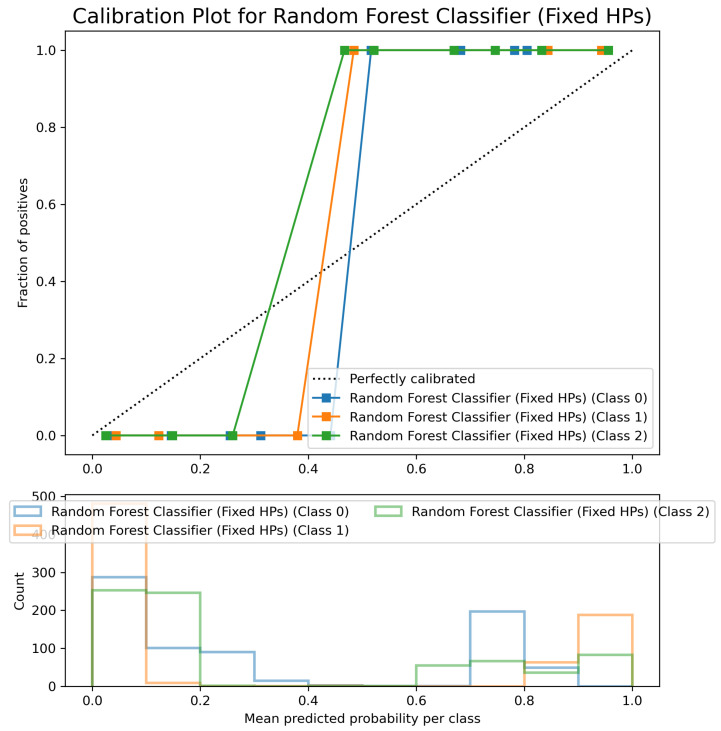
Calibration Plot for Random Forest Classifier (fixed hyperparameters). Classes are differentiated by colors.

The Random Forest Classifier’s Calibration Plot (Figure 19) shows reasonably well-calibrated predicted probabilities, with class curves following the diagonal line relatively closely, particularly for Classes 1 and 2. Class 0 shows slight underconfidence in lower probability ranges but maintains good calibration overall. This demonstrates reliable probability forecasts suitable for real-world applications requiring confidence estimations.

**Figure 20:**
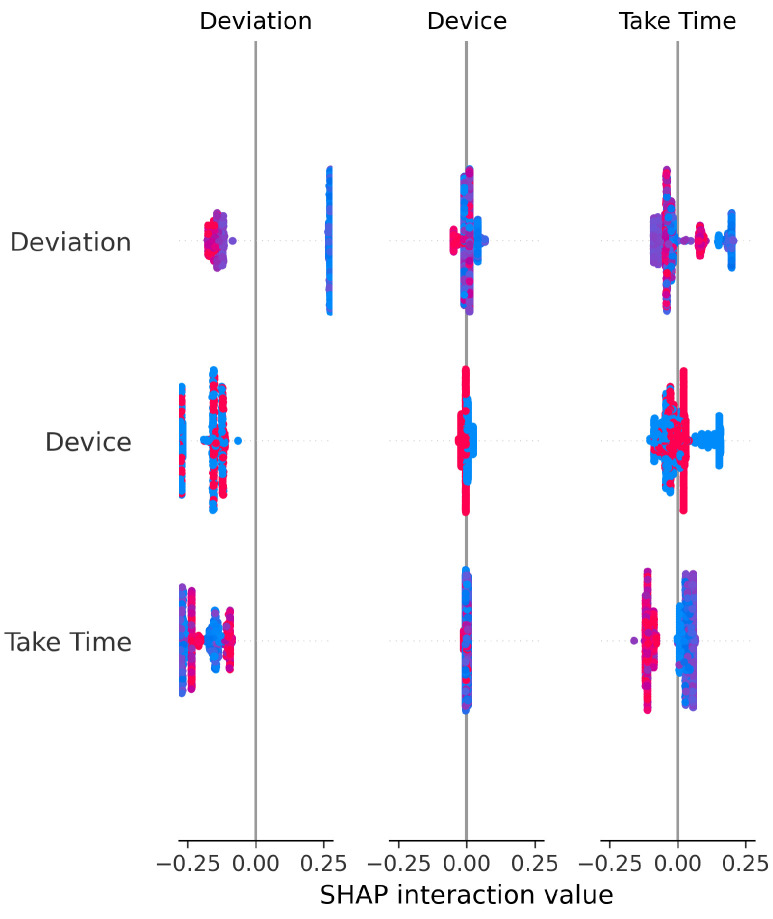
SHAP Summary Plot for Random Forest Classifier (fixed hyperparameters). High valued features are represented in red, while low valued features are represented in blue.

The SHAP Summary Plot for the Random Forest Classifier (Figure 20) confirms that Device and Deviation exert the most balanced and consistent influence across predictions. Their effects are primarily direct, rather than resulting from complex interactions with other variables.

RandomizedSearchCV’s Random Forest achieved a perfect CV score of 1.000 ± 0.000 with optimal hyperparameters: n_estimators = 221, max_depth = 24, max_features = 0.966, min_samples_split = 8, min_samples_leaf = 8, criterion = ’gini’.

### 5.4. Kernel Function Evaluation

To comprehensively assess model complexity requirements beyond the linear kernel, SVM models with Radial Basis Function (RBF) and Polynomial kernels were evaluated through systematic hyperparameter optimization.

#### 5.4.1. SVM RBF Kernel Performance

**Figure 21:**
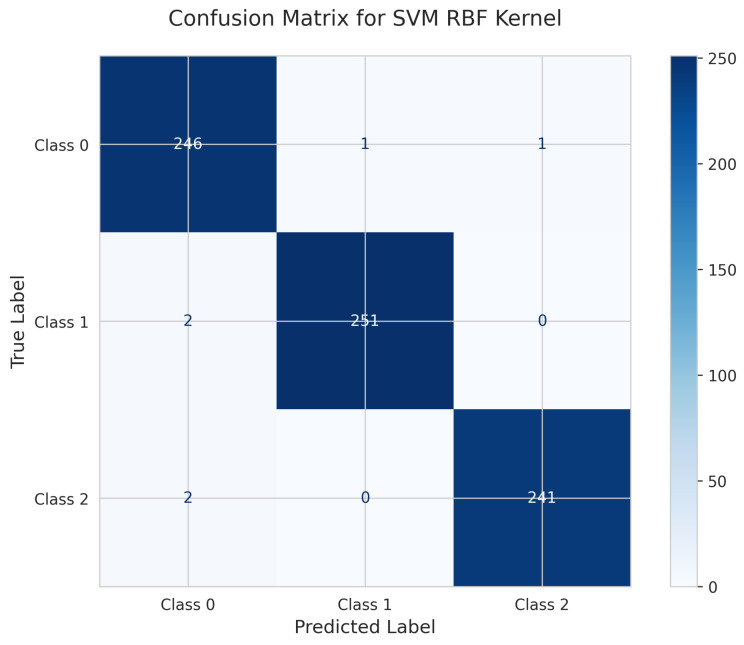
Confusion Matrix for SVM RBF Kernel.

The SVM with RBF kernel’s Confusion Matrix (Figure 21) shows 246 correct predictions for Class 0, with 1 misclassified as Class 1 and 1 as Class 2. Class 1 achieved 251 true positives with 2 misclassified as Class 0. Class 2 had 241 correct predictions with 2 misclassified as Class 0. The model achieved a test accuracy of 0.9919 (99.19%) with 6 total misclassifications.

**Figure 22:**
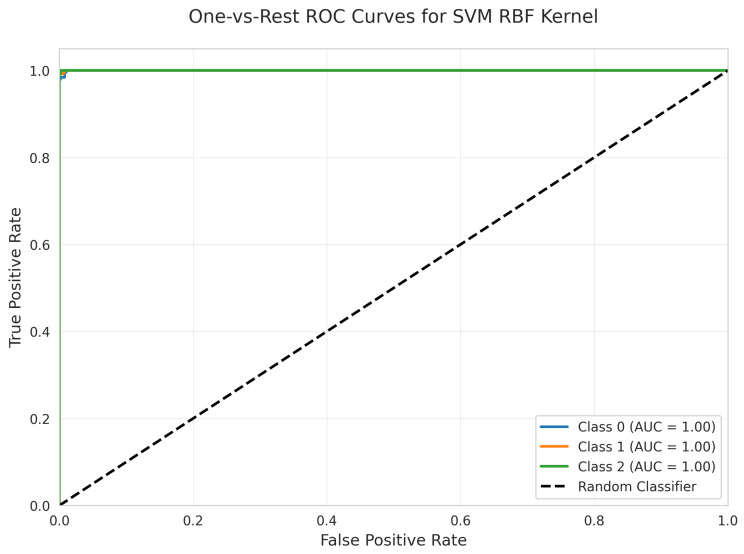
One-vs.-Rest ROC Curves for SVM RBF Kernel. All classes overlap at 100%.

The One-vs.-Rest ROC Curves (Figure 22) demonstrate that all classes achieve a perfect AUC of 1.00, indicating excellent discriminative ability despite the misclassifications in the confusion matrix.

**Figure 23:**
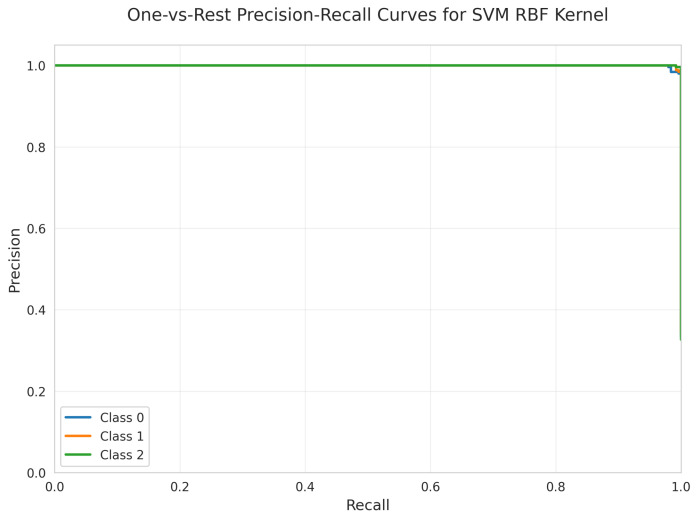
One-vs.-Rest Precision-Recall Curves for SVM RBF Kernel. All classes overlap at 100%.

The Precision-Recall Curves (Figure 23) show near-perfect precision across all recall values for all three classes, maintaining precision near 1.00 throughout most of the curves.

**Figure 24:**
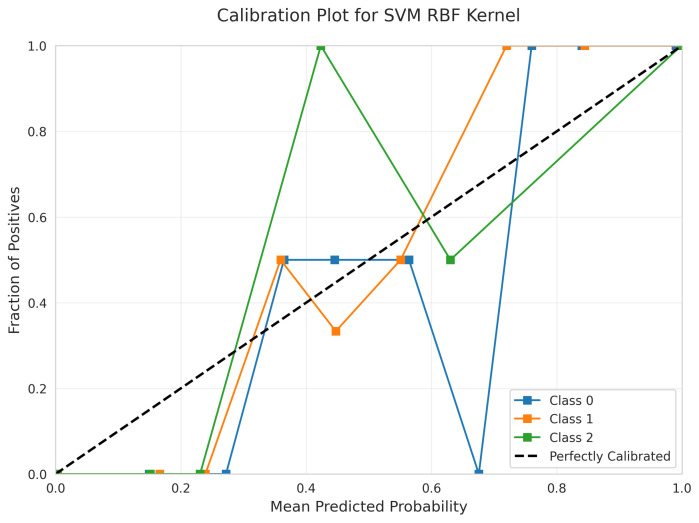
Calibration Plot for SVM RBF Kernel.

However, the Calibration Plot (Figure 24) reveals significant calibration issues. The curves exhibit erratic behavior with sharp discontinuities and unpredictable oscillations, particularly visible in the jagged patterns across all three classes. This indicates that the predicted probabilities are unreliable for confidence-based decision making, despite the model’s reasonable accuracy.

**Figure 25:**
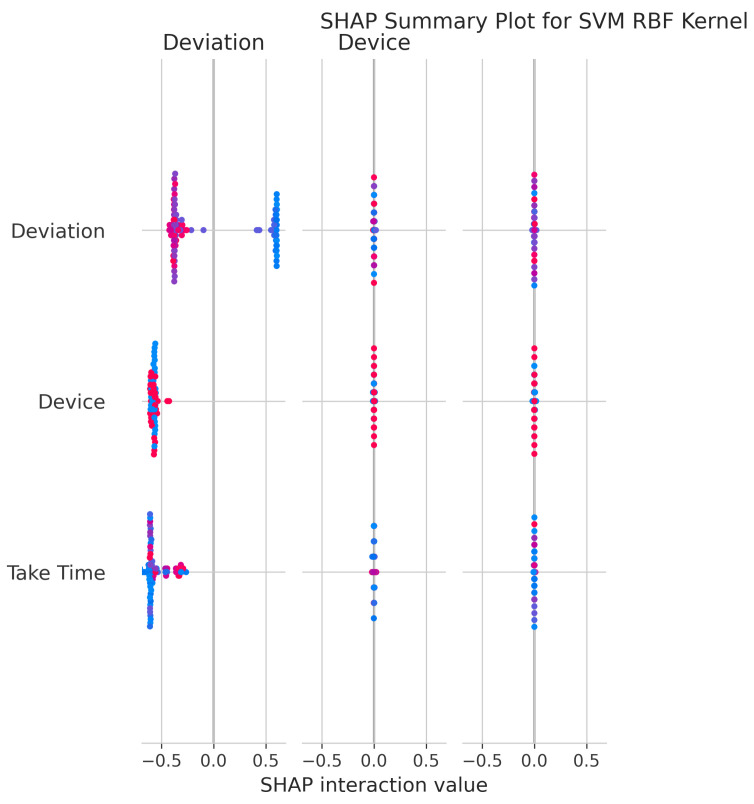
SHAP Summary Plot for SVM RBF kernel. High valued features are represented in red, while low valued features are represented in blue.

The SHAP Summary Plot (Figure 25) shows that Deviation remains the dominant feature, with Device and Take Time also contributing significantly to predictions.

RandomizedSearchCV identified optimal parameters: C = 65.41, gamma = 0.0035, class_weight = ’balanced’, achieving a best CV score of 0.9946 ± 0.0029. The RBF kernel introduced unnecessary complexity for this dataset, as evidenced by its poor calibration and lower accuracy compared to the linear kernel.

#### 5.4.2. SVM Polynomial Kernel Performance

**Figure 26:**
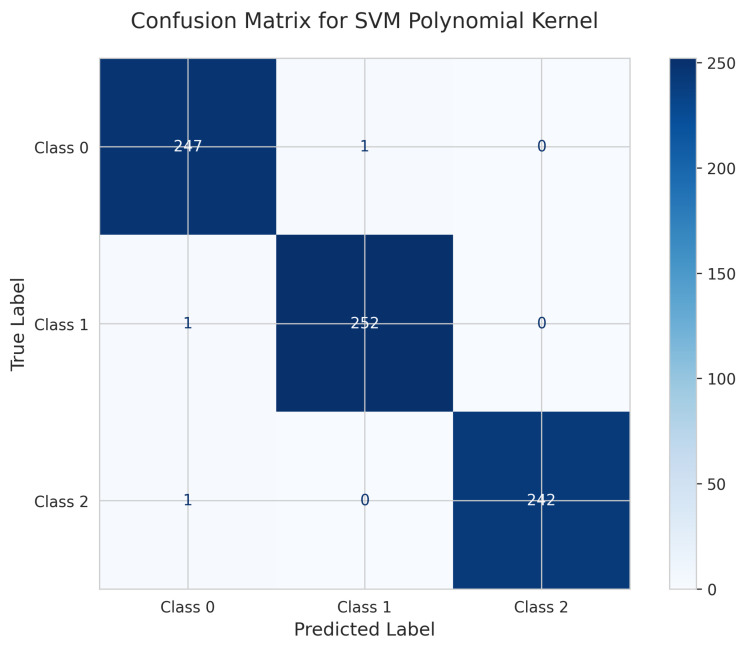
Confusion Matrix for SVM Polynomial kernel.

The SVM Polynomial kernel’s Confusion Matrix (Figure 26) demonstrates strong performance with 247 correct predictions for Class 0 (1 misclassified as Class 1), 252 true positives for Class 1 (1 misclassified as Class 0), and 242 correct for Class 2 (1 misclassified as Class 0). The model achieved a test accuracy of 0.996 (99.6%) with only 3 total misclassifications.

**Figure 27:**
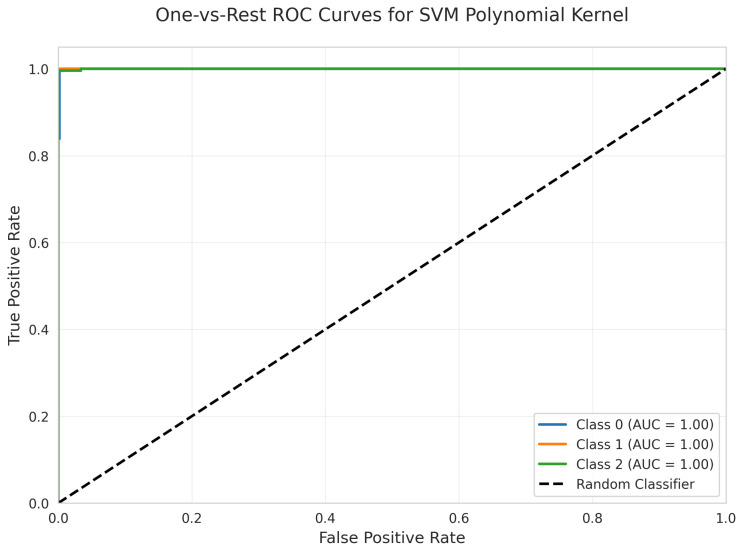
One-vs.-Rest ROC Curves for SVM Polynomial kernel. All classes overlap at 100%.

The One-vs.-Rest ROC Curves (Figure 27) show perfect AUC = 1.00 for all classes, confirming excellent discriminative capacity.

**Figure 28:**
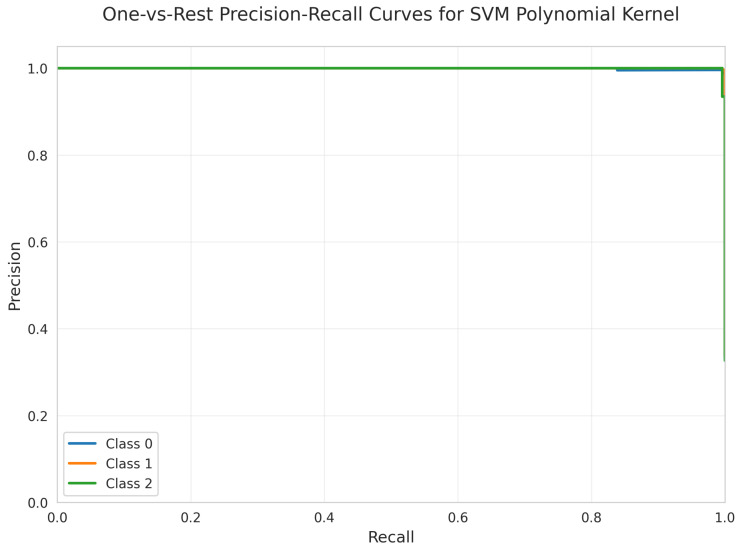
One-vs.-Rest Precision-Recall Curves for SVM Polynomial kernel. All classes overlap at 100%.

The Precision-Recall Curves (Figure 28) demonstrate near-perfect precision across all recall values for all classes, similar to the linear kernel’s performance.

**Figure 29:**
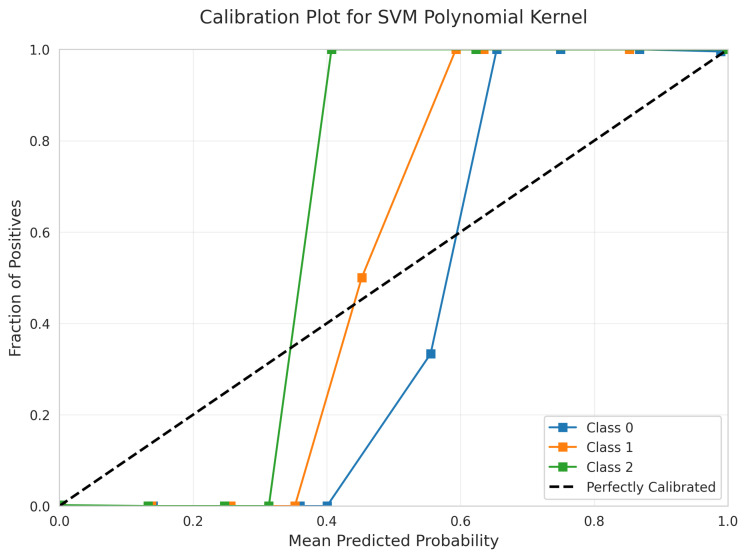
Calibration Plot for SVM Polynomial kernel.

The Calibration Plot (Figure 29) shows good calibration with curves following the diagonal relatively closely across most probability ranges, indicating reliable probability estimates.

**Figure 30:**
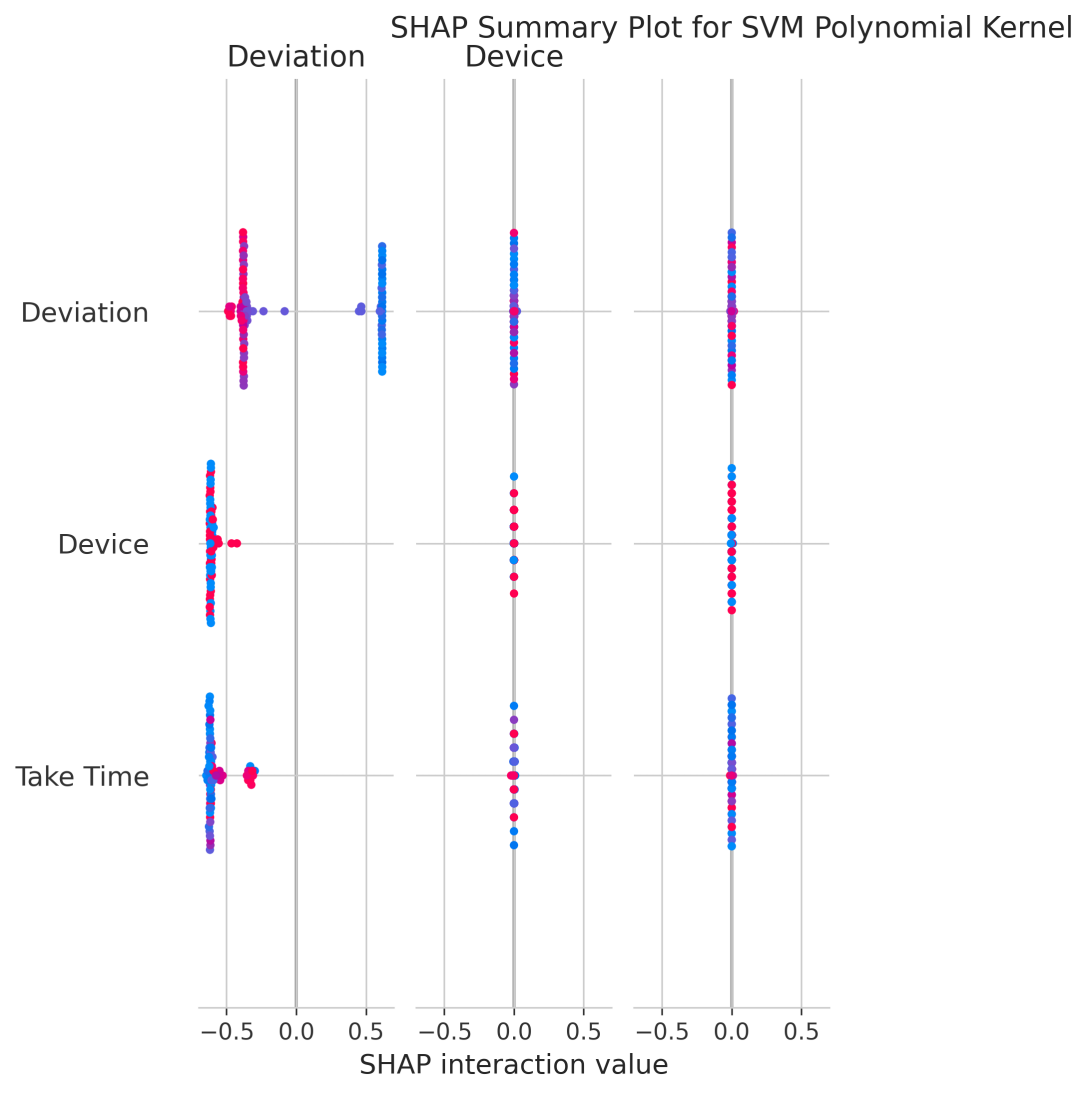
SHAP Summary Plot for SVM Polynomial kernel. High valued features are represented in red, while low valued features are represented in blue.

The SHAP Summary Plot (Figure 30) reveals that Deviation is the dominant feature, with Device and Take Time also contributing significantly to the model’s predictions.

RandomizedSearchCV identified optimal parameters: C = 34.17, degree = 2, gamma = 0.0576, coef0 = 0.271, achieving a best CV score of 0.9976 ± 0.0017.

### 5.5. Neural Network Performance

**Figure 31:**
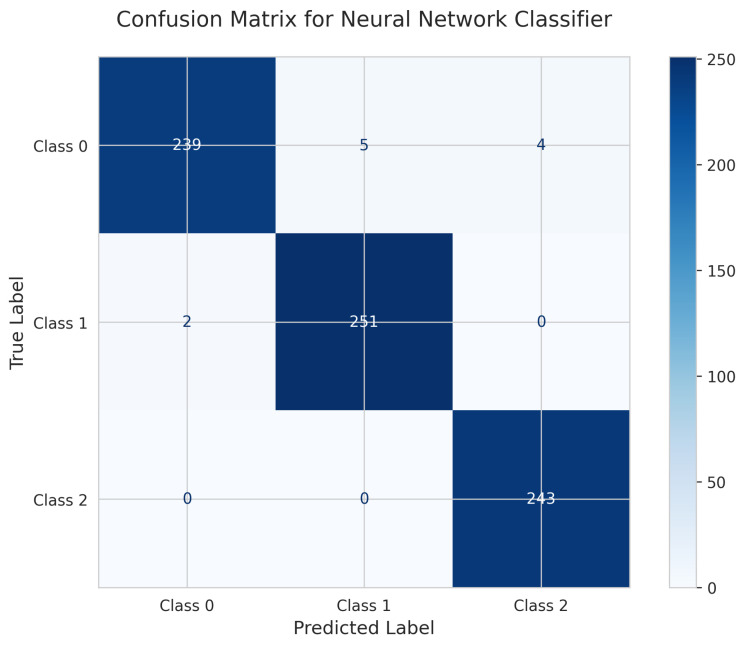
Confusion Matrix for Neural Network Classifier.

The Neural Network’s Confusion Matrix (Figure 31) shows 239 correct predictions for Class 0 (5 misclassified as Class 1, 4 as Class 2), 251 true positives for Class 1 (2 misclassified as Class 0), and 243 correct for Class 2 (all correctly classified). The model achieved a test accuracy of 0.9852 (98.52%) with 11 total misclassifications, making it the weakest performer despite being the most complex model.

**Figure 32:**
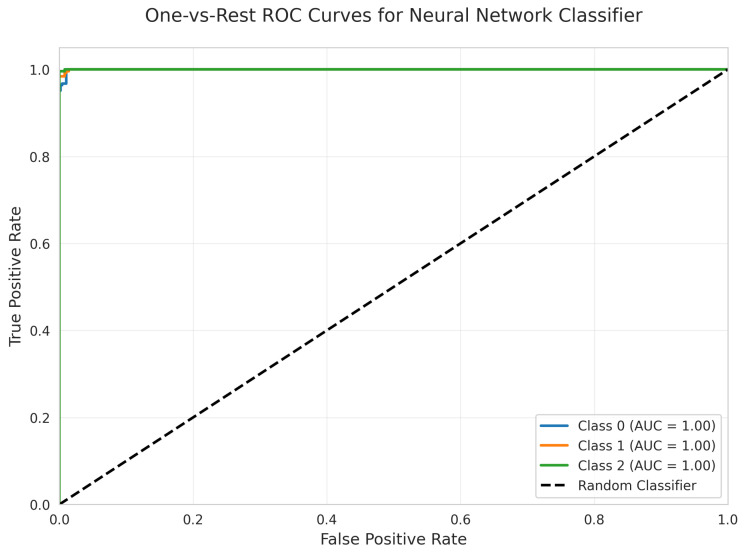
One-vs.-Rest ROC Curves for Neural Network Classifier. All classes overlap at 100%.

The One-vs.-Rest ROC Curves (Figure 32) show perfect AUC = 1.00 for all classes, indicating strong discriminative ability in terms of ranking predictions.

**Figure 33:**
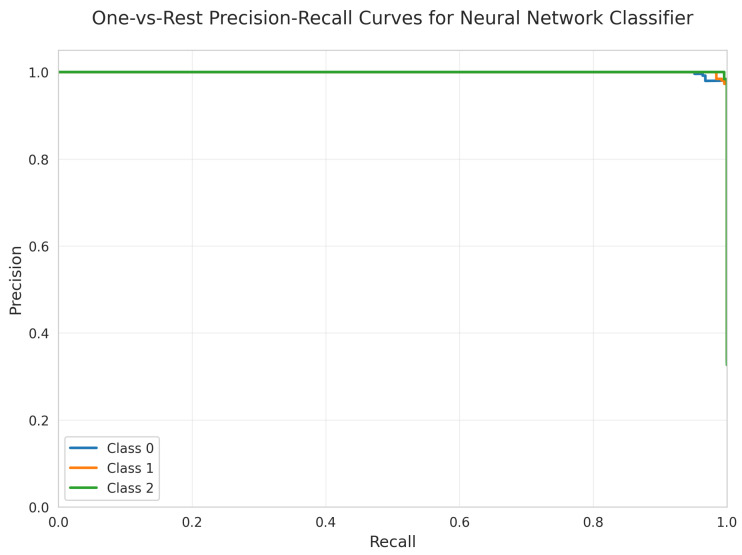
One-vs.-Rest Precision-Recall Curves for Neural Network Classifier. All classes overlap at 100%.

The Precision-Recall Curves (Figure 33) demonstrate near-perfect precision for Classes 1 and 2, with slightly lower performance for Class 0, consistent with the confusion matrix results.

**Figure 34:**
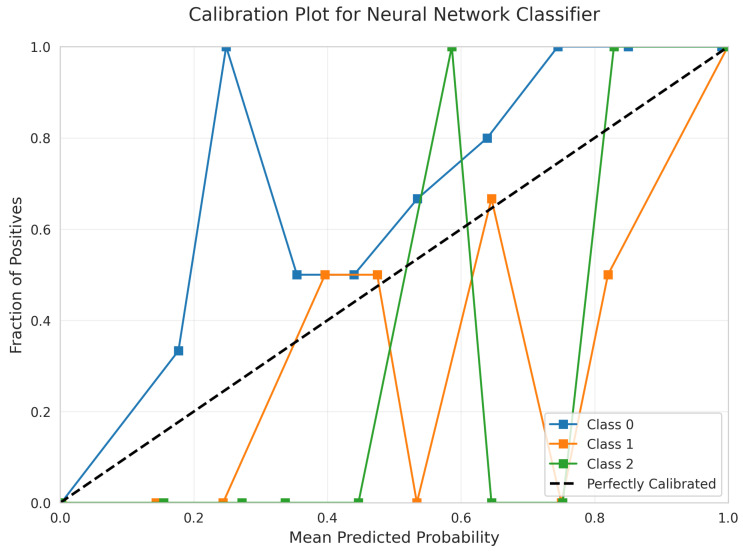
Calibration Plot for Neural Network Classifier. Classes are differentiated by color.

The Calibration Plot (Figure 34) reveals significant calibration problems with erratic curves that wildly deviate from the diagonal, showing multiple spikes and dips. This indicates unreliable probability estimates and suggests potential overfitting despite the model’s complexity.

**Figure 35:**
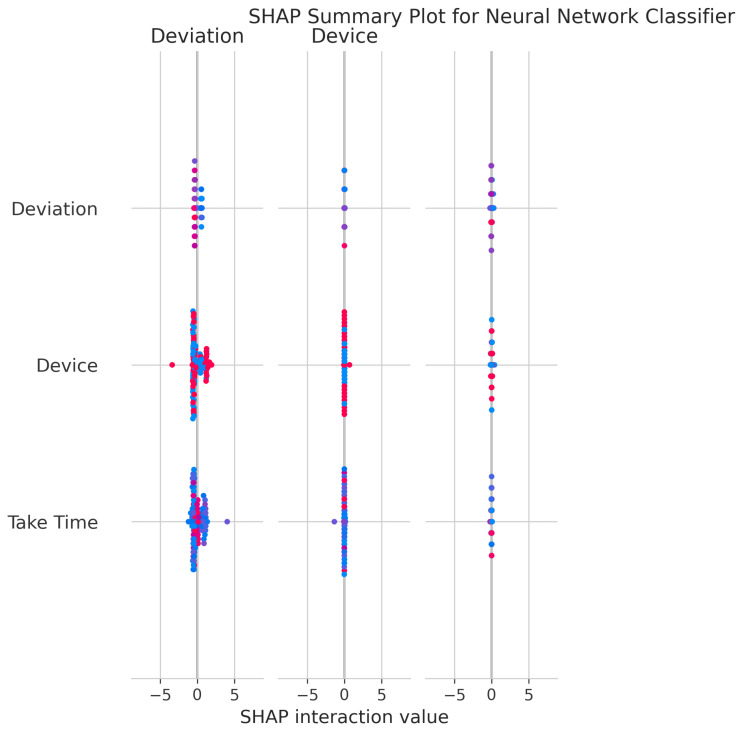
SHAP Summary Plot for Neural Network Classifier. High valued features are represented in red, while low valued features are represented in blue.

The SHAP Summary Plot (Figure 35) shows that Deviation and Device are the primary drivers of predictions, consistent with other models.

The Neural Network required substantially longer training time (20.54 s) with slower inference (0.36 ms) compared to simpler models, without providing any performance advantage.

### 5.6. Comparative Analysis of Model Performance

To comprehensively evaluate the classification approaches, model performance was assessed across multiple dimensions, including accuracy metrics, discriminative ability, probability calibration quality, computational efficiency, and cross-validation stability. This multi-faceted analysis enables identification of the optimal model architecture for deployment in real-time motion classification applications.

#### 5.6.1. Overall Performance Metrics (Accuracy, Precision, Recall, F1-Score)

Perfect Performance Tier: Both Random Forest (fixed HPs) and Logistic Regression achieved perfect test accuracy of 1.000 (100%) with zero misclassifications, as clearly demonstrated in their confusion matrices (Figure 16 and Figure 11), proving that the dataset exhibits strong linear separability.

Excellent Performance Tier: Linear SVM (99.6% with 1 error) and SVM Polynomial (99.6% with 3 errors) achieved excellent results with minimal misclassifications.

Good Performance Tier: SVM RBF (99.19% with 6 errors) and Neural Network (98.52% with 11 errors) performed adequately but were the weakest models despite higher complexity.

#### 5.6.2. Class-Wise Discrimination (ROC AUC and Precision-Recall Curves)

All models achieved perfect ROC AUC = 1.00 for all classes (Figure 7, Figure 12, Figure 17, Figure 22, Figure 27 and Figure 32), indicating optimal discriminative ability. This consistency confirms that the classes are highly separable in the feature space. The Precision-Recall curves (Figure 8, Figure 13, Figure 18, Figure 23, Figure 28 and Figure 33) similarly demonstrated near-perfect performance across all models.

#### 5.6.3. Probability Calibration

Excellent Calibration: Linear SVM (Figure 9) and Logistic Regression (Figure 14) exhibited superior calibration with curves closely tracking the diagonal across all probability ranges.

Good Calibration: Random Forest (Figure 19) and Polynomial SVM (Figure 29) showed good calibration with minor deviations.

Poor Calibration: Neural Network (Figure 34) and SVM RBF (Figure 24) demonstrated poor calibration with erratic curves and sharp discontinuities, making them unsuitable for applications requiring reliable probability estimates despite reasonable accuracy scores.

#### 5.6.4. Comprehensive Kernel Function Comparison

Table 3 presents a comprehensive comparison of SVM kernel functions to evaluate whether non-linear decision boundaries provide advantages over linear separation.

The linear kernel achieved equal or superior performance to non-linear alternatives across all metrics. Most significantly, it outperformed the RBF kernel in test accuracy (99.6% vs. 99.19%), made fewer errors (1 vs. 6), and demonstrated better cross-validation stability (±0.001 vs. ±0.003). The RBF kernel’s lower accuracy and poor calibration demonstrate that added complexity does not improve performance for this linearly separable dataset.

Linear Separability Confirmation: The superior performance of the linear kernel provides empirical evidence that the engineered feature space exhibits linear separability. When a linear decision boundary achieves optimal separation, more complex non-linear boundaries risk overfitting to noise rather than capturing genuine class structure.

Feature Engineering Validation: Successful linear separation indicates that the feature engineering process effectively transformed raw IMU sensor data into a representation where motion classes occupy linearly separable regions of the feature space.

Computational Efficiency: The linear kernel required 39% less training time (0.14 s vs. 0.23 s for RBF) and 80% faster inference (0.01 ms vs. 0.05 ms), critical advantages for real-time motion classification applications.

#### 5.6.5. Cross-Validation Stability Analysis

All models were evaluated using 5-fold cross-validation aligned with the 80/20 train-test split ratio. Table 4 demonstrates the stability and reliability of performance estimates.

The extremely low standard deviations (≤0.003 across all models) and minimal coefficients of variation (<0.3%) confirm that performance metrics are not artifacts of fortunate data partitioning but represent genuine model capabilities. Random Forest achieved zero variance across all folds (CV = 1.000 ± 0.000), indicating perfect consistency.

#### 5.6.6. Feature Importance and Interpretability

The consistent importance of Deviation, Take Time, and Device is evident across all models through both coefficient-based importance and SHAP analysis (Figure 10, Figure 15, Figure 20, Figure 25, Figure 30 and Figure 35). Linear models emphasize Deviation and Take Time most strongly, while tree-based models show more balanced importance, including Device. These features capture the most important information about the motion states. The visual plots demonstrate that their influence is primarily direct rather than highly dependent on intricate interactions with other minor features.

#### 5.6.7. Computational Efficiency

Linear SVM demonstrated the highest computational efficiency with training time of 0.14 s and inference time of 0.01 ms per sample. Logistic Regression showed training time of 0.91 s with 0.01 ms inference. Random Forest required 0.27 s training and 0.02 ms inference. The Neural Network exhibited substantially higher computational cost (20.54 s training, 0.36 ms inference) without corresponding performance benefits. All top-performing models demonstrate excellent computational efficiency suitable for real-time deployment.

#### 5.6.8. Consolidated Performance Metrics

Table 5 presents a comprehensive summary of all evaluated models.

### 5.7. Key Findings and Performance Analysis

Model Selection: Both Logistic Regression and Random Forest achieved perfect 100% accuracy, as clearly demonstrated in their confusion matrices (Figure 11 and Figure 16). Logistic Regression demonstrates optimal performance due to faster training (0.91 s vs. 0.27 s) combined with perfect accuracy and excellent calibration. Random Forest serves as an excellent alternative if ensemble robustness is prioritized.

Linear Separability Validated: The comprehensive kernel comparison definitively shows that linear models match or outperform non-linear alternatives. The linear SVM achieved 99.6% accuracy versus 99.19% for RBF, with superior calibration (Figure 9 vs. Figure 24). This validates successful feature engineering that captures motion patterns in a linearly separable space.

Cross-Validation Confirms Robustness: 5-fold CV with extremely low standard deviations (≤0.003, Table 4) proves that results represent genuine model capabilities rather than fortunate data splits. Random Forest’s zero variance (1.000 ± 0.000) demonstrates perfect consistency across all folds.

Kernel Function Evaluation: Three distinct SVM kernels were systematically evaluated through hyperparameter optimization (Table 3). The linear kernel demonstrated superiority across all metrics: fewest errors (1 vs. 3–6), best calibration quality, fastest training and inference times, and highest CV stability. Despite exhaustive hyperparameter searches (20 candidates for RBF and Polynomial), non-linear kernels could not surpass linear performance.

Calibration Quality Assessment: Only Linear SVM (Figure 9), Logistic Regression (Figure 14), and Random Forest (Figure 19) provide reliable calibrated probabilities. Neural Network (Figure 34) and RBF SVM (Figure 24) exhibited severely erratic calibration curves with sharp discontinuities, making them unsuitable for applications requiring confidence estimates despite adequate accuracy.

Model Complexity Trade-offs: The Neural Network, despite being the most complex model with the longest training time (20.54 s), achieved the lowest accuracy (98.52%) with the most errors (11), as shown in Figure 31. This demonstrates that unnecessary complexity degrades performance when the underlying data structure is simple and linearly separable.

Feature Importance Consistency: SHAP analysis across all models (Figure 10, Figure 15, Figure 20, Figure 25, Figure 30 and Figure 35) consistently identifies Deviation, Take Time, and Device as the most influential features. This information is essential for sensor selection and future feature engineering efforts.

Computational Efficiency: All top-performing models achieve sub-millisecond inference times suitable for real-time applications. Linear SVM offers the fastest training (0.14 s), while Logistic Regression and Random Forest balance speed with perfect accuracy.

## 6. Conclusions

This study presents an intelligent motion-tracking dental handpiece integrated with an inertial measurement unit (IMU) for real-time evaluation of tool movement. The system records metrics including roll, pitch, yaw, acceleration, velocity, Take Time, and Deviation, enabling precise assessment of operator performance.

The developed prototype successfully demonstrated real-time motion monitoring and classification of dental procedures using the newly collected dataset comprising 3720 records from 61 practitioners. This dataset captures realistic hand motion dynamics during cavity preparation and represents one of the first structured data resources for AI-based dental motion analysis.

Among the various classification models evaluated, both Logistic Regression and Random Forest classifiers exhibited the best performance, attaining perfect 100% accuracy and flawless AUC scores of 1.00 across all categories with zero misclassifications. Linear SVM closely followed, achieving 99.6% accuracy with only one misclassification and nearly perfect weighted AUC. The comprehensive kernel comparison revealed that the linear kernel outperformed non-linear alternatives, with the RBF kernel, achieving 99.19% accuracy (six errors) and the Polynomial kernel matching the Linear SVM at 99.6% (three errors).

Feature importance analysis consistently identified Deviation and Take Time as the most influential predictors across all models, underscoring their critical role in evaluating motion states. Device type also contributed substantially, particularly within tree-based models. These results confirm the system’s effectiveness in analyzing user dexterity and control, demonstrating its significant potential as a tool for dental education and training.

In conclusion, the Intelligent Dental Handpiece (IDH) bridges the gap between manual skill assessment and automated performance evaluation by embedding sensing, analysis, and feedback directly into a conventional dental tool. The findings validate the feasibility of integrating AI-driven motion analytics into dental education and clinical training.

### Future Work

To enhance model robustness, future research will expand the dataset to include a broader range of users and scenarios. Implementing real-time feedback methods during procedures may further improve training outcomes. Clinical validation will be essential to assess the system’s practicality and generalizability in dental practice.

Moreover, while the proposed intelligent handpiece demonstrates high accuracy in classifying motion states based on IMU-derived features such as acceleration, velocity, and orientation, it does not currently capture other important parameters that influence procedural quality, such as applied pressure on dental tissue. Applied pressure is important in assessing both dexterity and safety during dental procedures, as excessive force may lead to patient discomfort or tissue damage. Future enhancements to the IDH could integrate force sensors or pressure-sensitive overlays to capture this dimension, enabling a more comprehensive assessment framework.

## Figures and Tables

**Table 1:** Comparison of commercial dental simulation tools.

Tool Name	Key Features	Target Applications	Haptics Enabled
Simodont Dental Trainer [19,20]	High-fidelity 3D virtual simulation, realistic tactile feedback, performance tracking, wide library of clinical scenarios	Cavity preparation, crown prep, endodontics, preclinical skill development	Yes
Virteasy Dental (HRV Simulation) [21]	Immersive VR training, customizable scenarios, scoring system	Operative dentistry, prosthodontics, implantology	Yes
Moog Simodont (Legacy) [22]	Precision haptics, touchscreen interface, adjustable tools	Basic dental training, skill acquisition	Yes
IDEA Dental Handpiece Simulator	Integrated motion tracking in handpiece, real-time feedback	Dexterity training, performance analysis in handpiece control	No (focuses on movement data)
Forsslund Systems Haptic Dental Trainer [23]	Dual-handed training, procedural simulation with haptic arms	Crown prep, caries removal, ergonomics training	Yes
PerioSim [24]	3D tooth and gum models, periodontal probing with force feedback	Periodontal diagnostics and treatments	Yes
VRDTS (Virtual Reality Dental Training System) [25]	Real-time 3D visuals, force feedback, performance tracking	Cavity preparation, caries removal, preclinical operative dentistry	Yes
IDSS (Intelligent Dental Simulation System) [26]	Haptic-enabled training with AI-based assessment, tissue feedback, and real-time scoring	Caries removal, crown prep, endodontics, general operative dentistry	Yes
Intelligent Dental Handpiece (IDH)	Real-time motion tracking, IMU-based feedback, machine learning classification, cloud analytics	Dexterity training, motion classification, skill assessment	No (focuses on motion sensing and feedback)

**Table 2:** Hyperparameter optimization configuration by model.

Model	Search Iterations	CV Folds	Parameters Tuned	Total Evaluations
Logistic Regression	20	5	3	100
Random Forest	30	5	6	150
SVM (Linear Kernel)	15	5	2	75
SVM (RBF Kernel)	20	5	3	100
SVM (Polynomial Kernel)	20	5	5	100
Neural Network	–	–	Fixed Arch.	–
Total Cross-Validation Runs:				525

**Table 3:** SVM kernel function performance comparison.

Kernel	Test Acc	CV Score	Train (s)	Infer (ms)	Errors	Calibration
Linear	99.6%	0.999 ± 0.001	0.14	0.01	1	Excellent
Polynomial	99.6%	0.998 ± 0.002	0.16	0.03	3	Good
RBF	99.19%	0.995 ± 0.003	0.23	0.05	6	Poor

**Table 4:** Cross-validation performance and stability.

Model	CV Mean	CV Std Dev	Coeff. of Variation
Random Forest	1.0000	±0.0000	0.00%
Logistic Regression	0.9987	±0.0007	0.07%
Linear SVM	0.9990	±0.0008	0.08%
SVM Polynomial	0.9976	±0.0017	0.17%
SVM RBF	0.9946	±0.0029	0.29%

**Table 5:** Consolidated performance metrics comparison—all models.

Metric	LR	RF	SVM-L	SVM-P	SVM-R	NN
Train (s)	0.91	0.27	0.14	0.16	0.23	20.54
Test Acc	1.000	1.000	0.996	0.996	0.9919	0.9852
Precision	1.000	1.000	0.996	0.996	0.9919	0.9852
Recall	1.000	1.000	0.996	0.996	0.9919	0.9852
F1	1.000	1.000	0.996	0.996	0.9919	0.9852
CV Score	0.9987 ± 0.001	1.000 ± 0.000	0.999 ± 0.001	0.998 ± 0.002	0.995 ± 0.003	N/A
Infer (ms)	0.01	0.02	0.01	0.03	0.05	0.36
Errors	0	0	1	3	6	11

LR = Logistic Regression, RF = Random Forest, SVM-L/R/P = Linear/RBF/Polynomial SVM, NN = Neural Network. All metrics reported are weighted averages across all three classes.

## Data Availability

The dataset is available at this link: https://doi.org/10.17632/h76rf38jkn.1 (accessed on 15 October 2025).
